# An in-silico study to design C_60_ fullerene-based nanosensors for the adsorption, detection, and removal of the narcotic drug γ-hydroxybutyric acid

**DOI:** 10.1038/s41598-026-40808-9

**Published:** 2026-02-23

**Authors:** Ruaa M. Almotawa

**Affiliations:** Department of Chemistry, College of Science, University of Hafr Al Batin, Hafr Al Batin, 31991 Saudi Arabia

**Keywords:** Density Functional Theory (DFT), Gamma-hydroxybutyric acid (GHB), Sensor Design, Drug Detection, Fullerene C_60_, Electrochemical sensor, Chemistry, Materials science, Nanoscience and technology

## Abstract

γHydroxybutyric acid (GHB), a depressant of the central nervous system, is commonly used illegally and in drug-facilitated crimes; therefore, it is crucial to develop reliable and fast methods for detecting GHB. This study uses DFT theory to design and evaluate the performance of electrochemical and colorimetric nanosensors based on fullerene and its forms of doping with boron and zinc for GHB detection. The calculation results (bond length, HOMO-LUMO energy gap, infrared spectra and UV-visible absorption spectra) for C_60_ showed very good overlap with experimental results in other literature, indicating the validity of the computational method used in this work. Several analyses (such as electronic structure calculations, adsorption energy evaluation, charge-transfer analysis, NBO, NCI/RDG, ELF, LOL, QTAIM, conductivity, recovery time, and optical response analyses) were performed to investigate the sensor performance. After comparing these results, Boron-Doped C_60_ (BC_59_) was found to be the best candidate for electrochemical sensing of GHB based on conductivity modulation & charge transfer behavior. In contrast, pure C_60_ with the largest spectral shift (in the visible range) was introduced as a suitable candidate for colorimetric measurement. Zinc-doped C_60_ adsorbs GHB best (based on adsorption properties), making it suitable for GHB removal and adsorption in purification applications. Overall, this computational study makes experimental efforts more targeted by qualitatively assessing sensor performance and reducing trial and error, and provides clear guidance for future experimental validation and development of efficient GHB detection platforms.

## Introduction

Gammahydroxybutyric acid (GHB) is a shortchain fatty acid and a depressant of the central nervous system that is used both medically and illicitly. Within the human body، GHB is present endogenously to a limited degree in the human brain، acting as a neurotransmitter and neuromodulator to modulate sleep and one’s ability to manage their emotions. Clinically، it is indicated at controlled dosages for narcolepsy and alCohol dependence، but it is also known as a “club drug” or “daterape drug” due to its euphoric and sedative effects when misused. The identification of GHB is paramount in forensic and clinical toxicology، where GHB could be quickly metabolized and has a limited detection time in biological specimens^1,3^. The ability to identify GHB in a comprehensive and timely manner will help ascertain cases of suspected drugfacilitated sexual assault، overdose، or poisoning، where GHB’s identification could be a vital piece of evidence. Analytical methods that include gas chromatographymass spectrometry (GCMS) and liquid chromatographymass spectrometry (LCMS) are considered sensitive for the identification and reliability quantification of GHB^4,5^.

Analytical methods like GCMS and LCMS have limits to availability and practicality، irrespective of being very accurate، because they require specialist manpower، an environment where temperature and pressure are controlled، costly tools and very high upkeep. We are now more than ever still looking for faster، cheaper، and onsite detection methods^6,7^.

To address this issue، colorimetric and electrochemical sensors have more than ever become popular as they are easier to use، inexpensive، and useful for rapid as well as on site detection systems^8,9^. The focus of these sensors is often some type of nanomaterial، or more specifically carbon based nanostructures، such as graphene، carbon nanotubes، and fullerenes، that have high surface area and excellent electrical and optical properties^10,11^. Fullerene C_60_، in particular، has shown excellent functionality because of its spherical structure، high electron affinity، and ability to engage in ππ interactions and charge transfer process. Paying special attention to C_60_ in particular is maintaining a balance، especially with πelectron transfer between these sensor and target molecules، that increases detection sensitivity and selectivity^12^.

In recent investigations, the use of fullerenes as diagnostic sensors has received increasing attention. For example، (A) Maleki et al. highlighted the excellent sensing performance of fullerene C_60_ and its associated derivatives for the detection of the anticancer drug dacarbazine^13^. Similarly، (B) K. Chagaleti et al. showed the potential of BN-doped heterofullerene C_60_ for the sensing of formaldehyde، as well as noninvasive diagnosis in breast cancer^14^. Likewise E. O. Okon et al. presented titanium and coppermodified fullerene C_60_ as a nanostructured sensor for the detection of toxic gaseous pollutants^15^. In all three studies، beyond demonstrating the inherent sensing ability of fullerene C_60_، it was also found that metal/heteroatomdoping enhanced the electronic، catalytic، and sensing properties، suggesting that these doped fullerenes may be quite promising as a sensor material.

The current study investigated the effectiveness of pure C_60_ and its Boron and Zinc Doping in detecting GHB, as Pure C_60_ has superior Sensing properties and as a result of the need to detect GHB. Bader et al. conducted a study of the use of C_24_ nanocluster to detect γ-butyrolactone (GBL) using DFT, and reported that C_24_ with Magnesium Doping has superior sensing capabilities to those with Beryllium and Calcium Doping. Although GBL is a common precursor to the faster converting of γ-hydroxybutyric acid (GHB) into the body, GBL and GHB have significantly different Electronic Structures, with respect to polarity, behaviour in solution, and Adsorption Behaviour; therefore, the two substances should not be compared to one another in regards to sensing ability. The structure of ultra small-sized C_24_ Nanoclusters differs from C_60_ in respect to curvature, level of π-Electron Delocalization, Physical Structure and how these materials can be practically utilized. The current experiment utilized Boron and Zinc Doped fullerenes (BC_59_ and ZnC_59_) as the basis of this experiment in order to assess how these dopants affected the Electrochemical Sensing and Irreversible Adsorption potentially resulting from the C_60_^16^. Consequently, this work provides complementary insights into the detection and removal of GHB using fullerene-based nanostructures (based on the agreement between the theoretical data in this work with experimental data from other literature for C_60_).

Regarding the selection of doping atoms, boron was chosen due to its electron-deficient nature, which may enhance charge transfer interactions and provide greater sensitivity to C_60_^17^. Meanwhile, zinc was considered as an element known to modify the electronic structure and subsequent catalytic activity of nanomaterials, thereby enhancing the adsorption of target molecules and the detection of C_60_ bound to the nanomaterials^18^.

The electronic properties of C_60_ have been reported to be enhanced by doping with boron and zinc in various literatures. For instance, KY Thajudeen et al. demonstrated that ZnC_59_ has high sensitivity, UV absorption red shift and recovery rate, all of which are paramount qualities for isobutyric acid (a biomarker for COVID-19) sensing^19^. H Hadi et al. also studied doped C_60_ with zinc to improve reactivity and sensitivity of methamphetamine, a drug compound^20^. Similarly, T Yadav et al. stated that B-doped C_60_ (BC_59_) was a terrific performer in the detection of epinephrine^21^. It is worth noting that alternative dopants, such as silicon, have also been reported to significantly modify the electronic properties of fullerene-based nanostructures. Si-doped C₆₀ systems introduce distinct covalent bonding characteristics and charge redistribution patterns compared to boron- and metal-doped analogues. A systematic investigation of Si-doped C₆₀ for GHB detection could therefore provide valuable comparative insight into periodic trends and dopant-dependent sensing mechanisms. Such an investigation, however, is beyond the scope of the present work and will be considered as an interesting direction for future studies^22^.

Due to the difficulties and high cost of producing nanosensor, there has been an increased use of Computational Modeling to help design nanosensor and determine how to tailor the material properties before manufacturing, thus lowering the expense while improving the methodology of testing. This study utilized Density Functional Theory (DFT) and Quantum Theory of Atoms in Molecules (QTAIM) to evaluate the interaction between GHB and C_60_-based nanostructures^23,24^. We believe that the results of this study, as an important step towards the effective development of GHB detection platforms, can help in the synthesis and experimental validation of these nanosensors in the future.

## Computational method

**Fig. 1:**
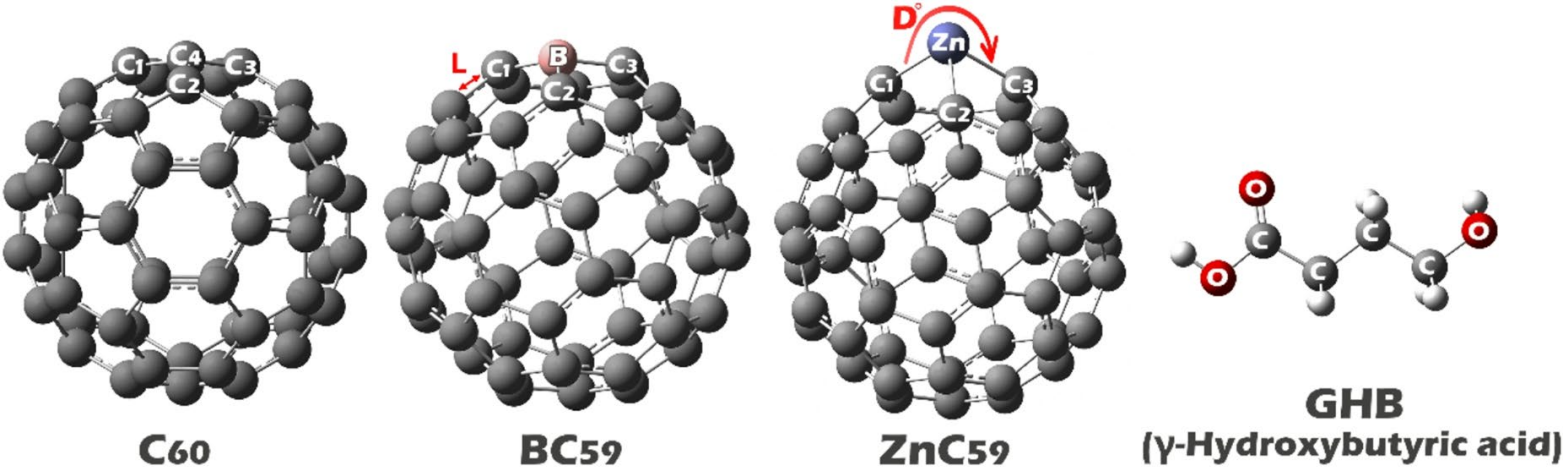
Structures studied in this work.

All structures (C_60_, BC_59_, ZnC_59_, GHB, C_60_@GHB, BC_59_@GHB, and ZnC_59_@GHB) were designed in GaussView 6 software (Version 6.0.16) (https://gaussian.com/gaussview6/) and their geometries were optimized using Gaussian 09 W software (Version B.01) (https://gaussian.com/glossary/g09/) (using density functional theory (DFT) at the B97D/6-31G* computational level) (Fig. 1)^25^. The geometric optimization was performed based on the default Gaussian convergence criteria. The _max_imum force (≤ 7.0 × 10^− 6^ a.u.), RMS force (≤ 1.0 × 10^− 6^ a.u.), _max_imum displacement (≤ 1.13 × 10^− 3^ Å) and RMS displacement (≤ 1.08 × 10^− 4^ Å) were used.

Water is the primary solvent in the bodies of humans and other biological systems. Nearly every chemical reaction within the body (including enzymes interacting with substrates as well as drugs binding to their targets) takes place in water, so it is important to accurately model how drugs and sensors would behave in water during an interaction. As such, the entire optimization process was carried out under aqueous conditions via the CPCM method (Conductor-like Polarizable Continuum Model, CPCM)^26^.

The B97D functional was selected because it accurately models non-covalent interactions like dispersion forces and offers users both good computational speed and accuracy when dealing with systems that involve weak interactions^27^. In addition, Avramopoulos and others showed that the electronic properties calculated for C_60_ (for example, the energy gap) by using the B97D functional have a high degree of correlation with reported experimental values^28^.

Finally, frequency calculations were also performed at the same theoretical level and no imaginary frequencies were observed, indicating local minima on the potential energy surface, confirming that all structures are stable and not in a transition state.

The Cohesive energy (E_Coh_) for each structure was calculated using Eq. 1.

1 $$\:{\boldsymbol{E}}_{\boldsymbol{C}\boldsymbol{o}\boldsymbol{h}}=\raisebox{1ex}{${\boldsymbol{E}}_{\boldsymbol{t}\boldsymbol{o}\boldsymbol{t}\boldsymbol{a}\boldsymbol{l}}-\sum\:{\boldsymbol{E}}_{\boldsymbol{a}\boldsymbol{t}\boldsymbol{o}\boldsymbol{m}}$}\!\left/\:\!\raisebox{-1ex}{$\boldsymbol{n}$}\right.$$

In this equation, E_total_: The total energy of the molecule when it is in its optimized form; ∑_atoms_: The sum of the energies of the individual atoms in the structure if they were isolated; n: the total number of atoms in the molecule^29^.

The most important reactivity parameters such as energy gap ($$\:\mathrm{H}\mathrm{L}\mathrm{G}=\left|{\mathrm{E}}_{\mathrm{H}\mathrm{O}\mathrm{M}\mathrm{O}}-{\mathrm{E}}_{\mathrm{L}\mathrm{U}\mathrm{M}\mathrm{O}}\right|$$), chemical hardness ($$\:{\upeta\:}=\raisebox{1ex}{$(-{\mathrm{E}}_{\mathrm{H}\mathrm{O}\mathrm{M}\mathrm{O}}-(-{\mathrm{E}}_{\mathrm{L}\mathrm{U}\mathrm{M}\mathrm{O}}\:\left)\right)$}\!\left/\:\!\raisebox{-1ex}{$2$}\right.$$), chemical softness ($$\:S=1/{\upeta\:}$$), fermi level $$\:\left({E}_{F}={E}_{HOMO}+\frac{\left({E}_{LUMO}-{E}_{HOMO}\right)}{2}\right)$$, work function ($$\:\varphi\:=-{E}_{f}$$) and chemical potential ($$\:{\upmu\:}=-(-{\mathrm{E}}_{\mathrm{H}\mathrm{O}\mathrm{M}\mathrm{O}}+(-{\mathrm{E}}_{\mathrm{L}\mathrm{U}\mathrm{M}\mathrm{O}}\left)\right)/2$$) were studied computationally. E_HOMO_ and E_LUMO_ represent the energy of the highest occupied molecular orbital and the lowest unoccupied molecular orbital, respectively^30,31^.

Charge transfer parameters such as _max_imum possible charge transfer (ΔN_max_), and electrophilicity-based charge transfer (ECT) were calculated using Eqs. 2 and 3, respectively. If ECT is negative, the sensor behaves as an electron acceptor. α and β are related to the ΔN_max_ of the sensor and the Sensor@Drug complex, respectively^32^.

2 $$\:{\varDelta\:\boldsymbol{N}}_{\boldsymbol{m}\boldsymbol{a}\boldsymbol{x}}=-\raisebox{1ex}{$\boldsymbol{\mu\:}$}\!\left/\:\!\raisebox{-1ex}{$\boldsymbol{\eta\:}$}\right.$$

3 $$\:\boldsymbol{E}\boldsymbol{C}\boldsymbol{T}={\left({\boldsymbol{\Delta\:}\boldsymbol{N}}_{\boldsymbol{m}\boldsymbol{a}\boldsymbol{x}}\right)}_{\boldsymbol{\alpha\:}}-{\left({\boldsymbol{\Delta\:}\boldsymbol{N}}_{\boldsymbol{m}\boldsymbol{a}\boldsymbol{x}}\right)}_{\boldsymbol{\beta\:}}$$

In order to evaluate the sensitivity of each sensor, the adsorption energy (E_ads_), recovery time (τ), and electrical conductivity (σ) were calculated for each of them using Eqs. 4–6.

4 $$\:{\mathbf{E}}_{\mathbf{a}\mathbf{d}\mathbf{s}}={\mathbf{E}}_{\left(\mathbf{Z}\mathbf{n}/\mathbf{B}\right){\mathbf{C}}_{60}@\mathbf{D}\mathbf{r}\mathbf{u}\mathbf{g}}-\left({\mathbf{E}}_{\boldsymbol{D}\boldsymbol{r}\boldsymbol{u}\boldsymbol{g}}+{\mathbf{E}}_{\left(\mathbf{Z}\mathbf{n}/\mathbf{B}\right){\mathbf{C}}_{60}}\right)$$

5 $$\:\boldsymbol{\tau\:}={\boldsymbol{V}}_{0}^{-1}\times\:\mathbf{e}\mathbf{x}\mathbf{p}(-\frac{{\boldsymbol{E}}_{\boldsymbol{a}\boldsymbol{d}\boldsymbol{s}}}{{\boldsymbol{k}}_{\boldsymbol{B}}\boldsymbol{T}})$$

6 $$\:\boldsymbol{\upsigma\:}=\boldsymbol{A}{\boldsymbol{T}}^{3/2}{\boldsymbol{e}}^{(-\boldsymbol{H}\boldsymbol{L}\boldsymbol{G}/2{\boldsymbol{K}}_{\boldsymbol{B}}\boldsymbol{T})}$$

In Eq. 4, E((Zn/B)C_60_@GHB) is the energy of complex formation of each of the sensors (C_60_, BC_59_, and SiC_59_) with GHB. E_Drug_ and E(Zn/B)C_60_ represent the energy of GHB and each of the sensors in the isolated state, respectively. Equations 5 and 6 define V_0_ the effort frequency (typically ~ 10^12^ s^−1^), k_B_ the Boltzmann constant, T the temperature (298 K), and A the Richardson constant (6 × 10^5^ A.m^−2^.K^−2^)^33,34^. It should be observed that the estimated value of recovery time (τ) from Eq. 5 comes from an Arrhenius-like equation describing the ideal kinetic behavior based only on the adsorption energy. Calculated τ values ​​are interpreted as representing relative recovery behavior, not actual T measurements in the laboratory. For systems that are strongly bound, this procedure will often result in an excessive overestimation of τ, especially in the absence of environmental influences (e.g., heat, light exposure, or electrical bias).

The dipole moments (µ) and polarizabilities (α) for the sensors in the presence and absence of GHB were evaluated using Eqs. 7 and 8^35^.

7 $$\:\boldsymbol{\mu\:}=\sqrt{{\boldsymbol{\mu\:}}_{\boldsymbol{x}}^{2}+{\boldsymbol{\mu\:}}_{\boldsymbol{y}}^{2}+{\boldsymbol{\mu\:}}_{\boldsymbol{z}}^{2}}$$

8 $$\:\boldsymbol{\alpha\:}=\frac{1}{3}\left({\boldsymbol{\alpha\:}}_{\boldsymbol{x}\boldsymbol{x}}+{\boldsymbol{\alpha\:}}_{\boldsymbol{y}\boldsymbol{y}}+{\boldsymbol{\alpha\:}}_{\boldsymbol{z}\boldsymbol{z}}\right)$$

Finally, after performing DFT-based electronic and energetic analyses, all of the alternative analyses were performed using the converged DFT wavefunctions generated at the B97D/6-31G* level within a CPCM solvation model. Using the built-in NBO module of Gaussian 09 W, the NBO analysis was performed to evaluate donor-acceptor interactions and charge-transfer effects. The NCI/RDG, ELF and LOL analyses of electron density were extracted using Multiwfn software (Version 3.8) (http://sobereva.com/multiwfn/) to identify the nature of non-covalent interactions^36^. Electron density properties were performed at critical bond points using QTAIM theory and AIM2000 software (Version 2.0) (http://www.aim2000.de/)^37^. These calculations were performed to understand the molecular interactions between C_60_ (and its contaminated forms) and GHB, as well as to design a novel diagnostic sensor or adsorbent for the detection or removal of GHB.

## Results and discussion

### Structural properties

The geometric and structural properties of molecules and materials can be defined by the bond length (the distance between two atoms) and the bond angle (the angle between two bonded atoms about a given central atom). Doped materials can change the electronic, optical, and mechanical properties of molecules by introducing local structural distortions (caused by differences in atomic radius or charge between the host and doped atoms) that cause changes^38^. Therefore, computational studies were performed to determine the effect of doping on the structural properties. These results are summarized in Table 1.

**Table 1:** Calculated values ​​of bond lengths (L) and bond angles (D) between some important atoms in the designed structures.

Structure	Bond lengths (Å)	Bond angles (°)			
		Theoretical	Experimental		
C_60_	C_4_-C_1_	1.45	1.40 to 1.45^39^	C_1_-C_4_-C_2_	120.01
	C_4_-C_2_	1.40		C_1_-C_4_-C_3_	108.01
	C_4_-C_3_	1.45		C_2_-C_4_-C_3_	120.01
BC_59_	B-C_1_	1.53	-----------	C_1_-B-C_2_	118.46
	B-C_2_	1.55	-----------	C_1_-B-C_3_	118.46
	B-C_3_	1.55	-----------	C_2_-B-C_3_	106.26
ZnC_59_	Zn-C_1_	1.97	-----------	C_1_-Zn-C_2_	91.95
	Zn-C_2_	2.03	-----------	C_1_-Zn-C_3_	99.56
	Zn-C_3_	2.02	-----------	C_2_-Zn-C_3_	82.00

The data in Table 1 show that for C_60_ (pristine) the optimal bond lengths are approximately 1.40–1.45 Å, which closely resembles the optimal bond lengths measured using experimental methods. It has been established experimentally using X-ray diffraction of the solid fullerene that C_60_ contains two unique C-C Bonds (the shorter (6, 6) bonds having values in the range of 1.40 : 1.42 Å and the longer (5, 6) Bonds measuring 1.45 : 1.46 Å)^39^, confirming that the theoretical methods used to perform this study produce the same C₆₀ structure that has been reported in the literature, thereby validating the methods employed to characterize C_60_. The angles between bonds in C_60_ are measured between 108° − 120°, which corresponds to the spherical geometry of C_60_ and the presence of both pentagonal and hexagonal rings. As a result, there is a near symmetry of the geometry of C_60_.

The substitution of carbon with boron in the structure of BC_59_ results in an observable alteration of both the bond lengths and angles of the molecule. The B-C bonds are somewhat longer than the original C-C bonds in C_60_, measuring between 1.53 Å and 1.55 Å; this is due to the increase in size of the boron atom’s atomic radius compared to carbon, as well as the fact that boron has a weaker bond strength than carbon. As a result of the boron substitution, the bond angles surrounding the boron atom (118.46°, 118.46°, and 106.26°) exhibit a distortion from the ideal fullerene geometry and therefore demonstrate a localized strain effect caused by the dopant. The structural changes noted from this substitution indicate that the bonding network is slightly weakened and that the overall symmetry of the molecule has been somewhat diminished.

ZnC_59_ contains the most significant deviations. The bond lengths (Zn-C) are significantly longer than in C_60_ and BC_59_ (1.97–2.03) due to the larger size (atomic radius) of zinc, and they also indicate that the nature of bonding between zinc and carbon is more ionic or metallic than covalent. The bond angles (ZnC-C, C-Zn-C, and C-C-C) of ZnC_59_ (91.95°, 99.56°, and 82°) are significantly more distorted than those in C_60_ and BC_59_, indicating a pronounced localized geometric deformation of the fullerene. Overall, the effects of Zn doping will lead to more pronounced geometric strain and will disrupt the delocalized π-system of C_60_ to a greater extent than those associated with boron doping.

### Cohesive energy

**Fig. 2:**
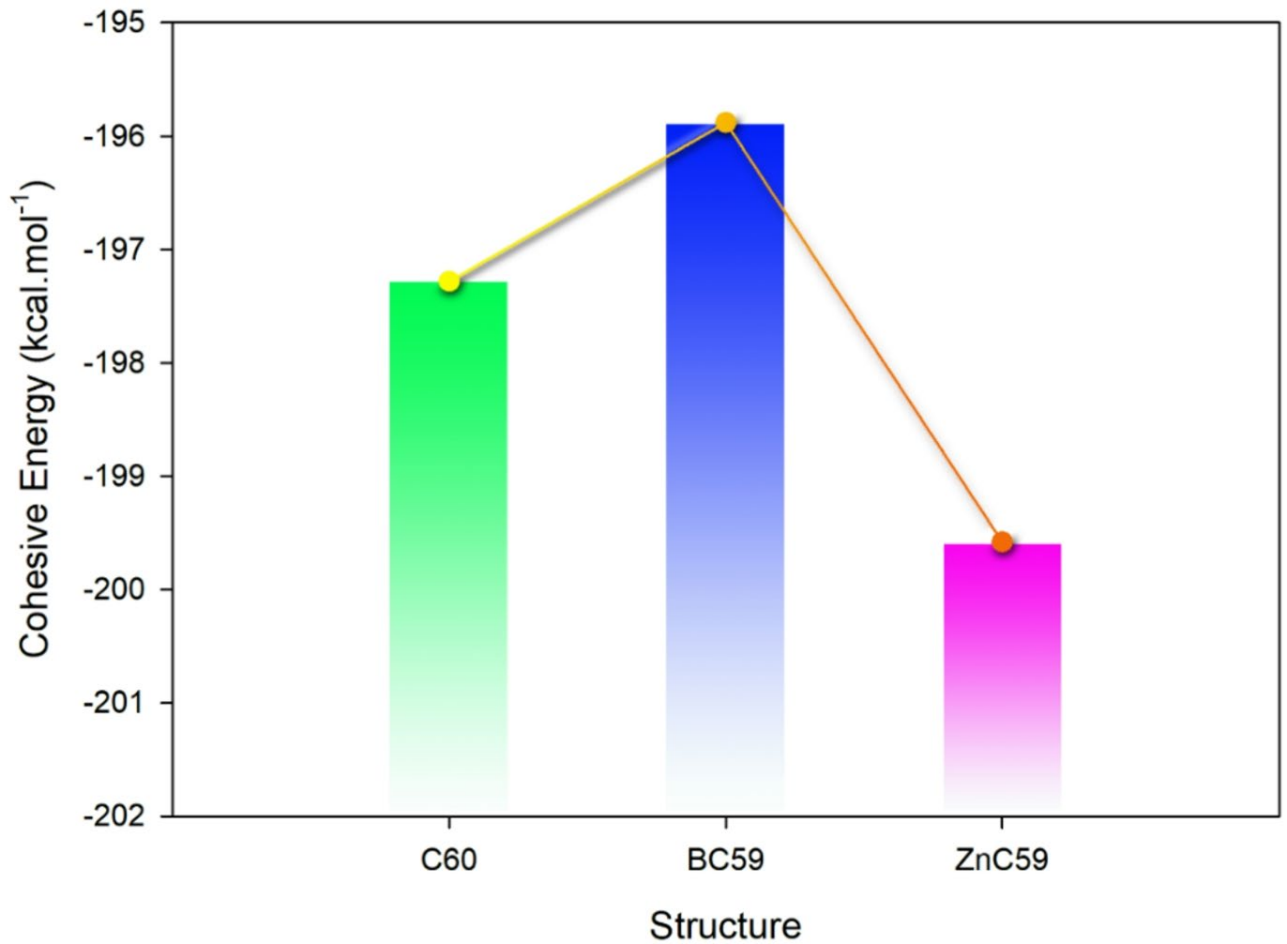
Qualitative trend of Cohesive energy changes for pristine C_60_ and its forms doped with B and Zn.

Cohesive energy (E_Coh_) is defined as the total energy required to separate a molecule or a solid into its individual atoms or molecules. The more negative the E_Coh_, the stronger the bonds within the material and the more resistant to dissociation. Doping as a strategy can affect the E_Coh_, so the effect of doping on the stability of C_60_ was studied computationally (see Fig. 2)^40^.

The E_Coh_ for C_60_ is −197.3 kcal/mol, which indicates that the carbon atoms in the fullerene structure are bonded well together. An E_Coh_ value of −197.3 kcal/mol can be used as a reference when comparing how doping with other elements influences the Cohesive energy of fullerene molecules. The BC_59_ has an E_Coh_ value of −195.9 kcal/mol, indicating that the bonding between carbon atoms in the BC_59_ molecule is somewhat weaker than that of pure C_60_ because of the addition of boron into the molecule. On the other hand, the ZnC_59_ has an E_Coh_ value of −199.6 kcal/mol, indicating that the addition of zinc to this fullerene has increased the strength of the bond between the carbon atoms.

### IR spectrum

**Fig. 3:**
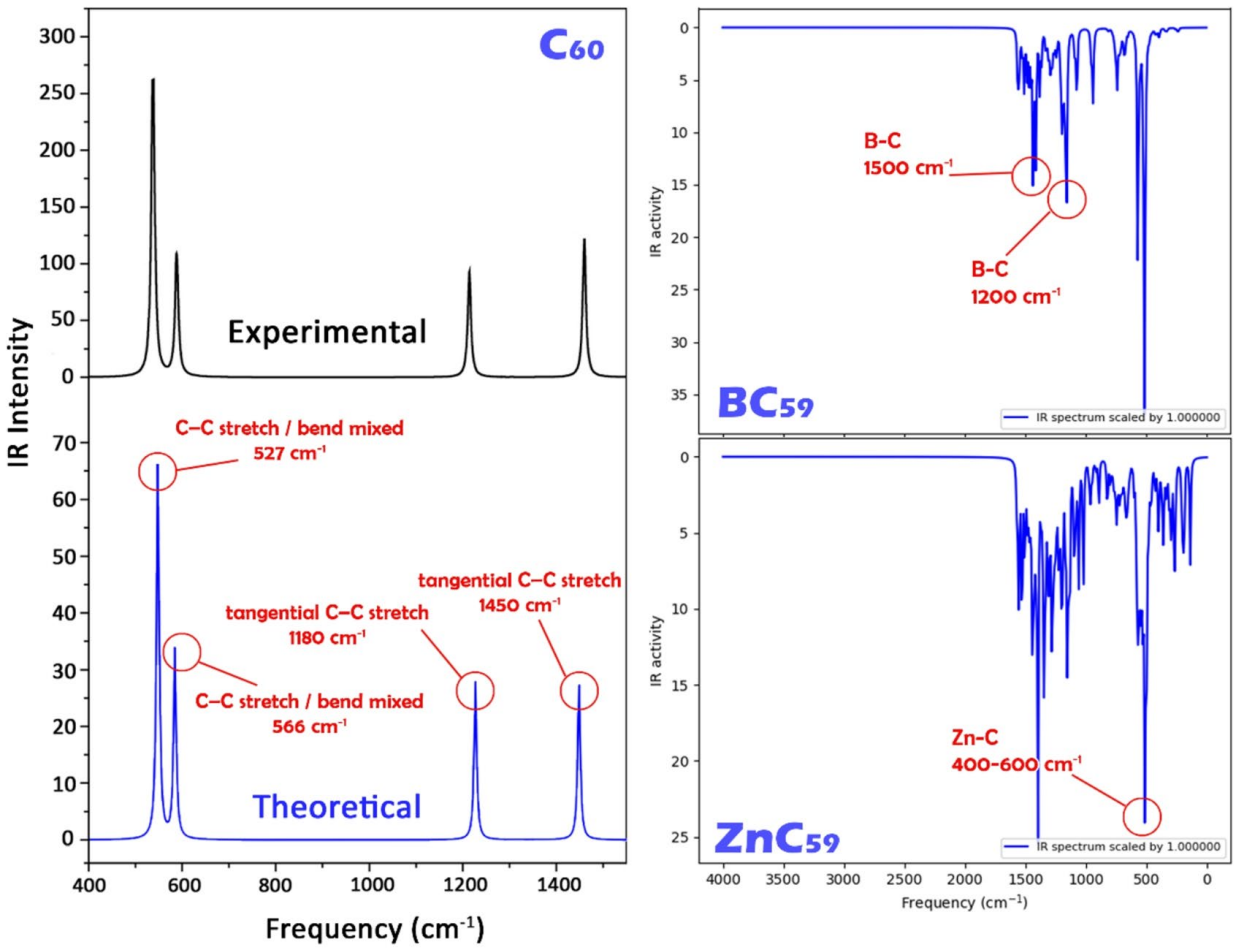
Experimental and theoretical IR spectra of pristine C_60_ and theoretical IR spectra of BC_59_ and ZnC_59_.

Analysis of theoretical infrared (IR) spectra combined with experimental evidence is a more accurate method for validating the structure of a designed molecule. When there is a good overlap between the experimental and theoretical IR spectra, it can be assumed that the designed molecule has similar properties to the synthesized compounds. If there is only a small degree of agreement, the IR spectrum may indicate a flaw in the synthesis or design^41^. For this reason, the IR spectra of all three sensors (C_60_, BC_59_, ZnC_59_) were calculated and subsequently compared with the experimental spectra (for C_60_) available in the literature (see Fig. 3).

The IR spectra of the C_60_, BC_59_, and ZnC_59_ systems can be found in Fig. 3. On the left-hand side, the experimental and computational spectra from the C_60_ system can be compared side-by-side. The pair of spectra allows the validation of the computational methods used to generate the spectra. In the case of the doped C_60_ systems, theoretical IR spectra were used to identify the vibrational features that result from the dopants.

The experimental IR spectrum for the C_60_ system closely matches the calculated IR spectrum that was derived by employing the B97D/6-31G* methodology. The C_60_ molecule, which has a high symmetry (Ih), is characterized by exhibiting only 4 IR-active fundamental vibrational modes. All of the vibrational modes that were produced experimentally are clearly represented in the theoretical IR spectrum.

The band found experimentally at 527 cm^− 1^ is identified as being caused by a mixed C-C stretching/bending mode related to the deformation of the radius of the fullerene structure. Theoretical calculations predict this same mode near 566 cm^− 1^, which is shifted slightly to the blue end of the spectrum (a very normal result for harmonic DFT calculations). The second experimental feature (itself at roughly 576 cm^− 1^) is similarly located in the same theoretical area as that predicted by computational methods, thereby providing further confirmatory evidence validating the nature of these two low-frequency vibrations.

In relation to the mid-frequency bands, the experimental band located at 1183 cm^− 1^ has been assigned to the tangentially-stretching vibration of C-C bonds along the side of the graphene structure. The computed spectrum predicts this peak at about 1180 cm^− 1^; thus, it confirms a very close correlation in both position and relative intensity between the two methods of analysis. This also holds true for the strongest experimental peak (located between 1429 and 1450 cm^− 1^); this peak represents the predominant mode of tangential C-C bond stretching in the case of C_60_, as such, it has also been accurately reproduced within the computed spectral data at around 1450 cm^− 1^.

The substitution of a carbon atom with a boron atom in B_59_C differs from C_60_ by the lowering of the symmetry of the cage and the consequent activation of additional IR bands. The BC_59_ IR spectrum is much more complex than that of C_60_, with IR bands appearing in the approximately 1200–1350 cm^− 1^ region due to the presence of B-C stretching vibrations coupled to C-C modes in the adjacent environment. IR bands appearing at approximately 1500 cm^− 1^ arise from perturbed tangential C-C stretching that results from electronic density displacement around the boron site.

The splitting and broadening of peaks in BC_59_ when compared to C_60_ are a direct result of symmetry breaking and the redistribution of electronic charge density caused by boron incorporation. The changes in the spectra of B_59_C relative to C_60_ provide strong evidence that boron incorporation into the fullerene will have a significant effect on the vibrational properties of the fullerene.

The ZnC_59_ spectrum (lower right panel) exhibits the most pronounced deviation from pristine C_60_. In addition to perturbed C-C stretching modes in the ~ 1000–1500 cm^− 1^ region, a strong and distinct absorption appears in the ~ 400–600 cm^− 1^ range. This band is characteristic of Zn-C stretching and metal-cage deformation modes, arising from the heavy atomic mass of zinc and the partially coordinative nature of the Zn-C interaction^43^.

The enhanced intensity and red-shifted low-frequency vibrations indicate strong coupling between the Zn center and the fullerene framework. These features are absent in pristine C_60_ and BC_59_, clearly identifying Zn incorporation and confirming the formation of a stable metal-fullerene interaction.

### MEP analysis

**Fig. 4:**
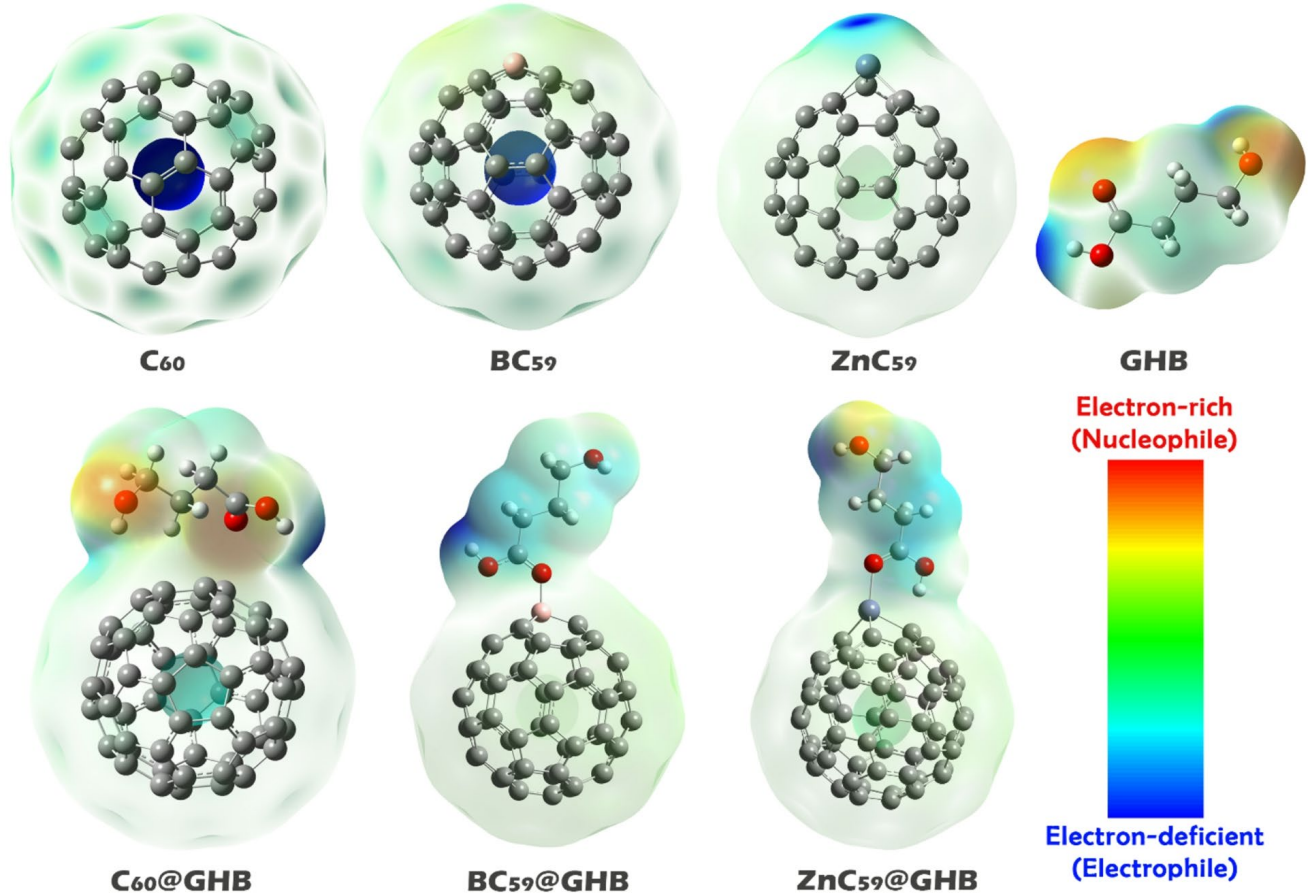
MEP contours for each of the instruments studied in this work.

Using the molecular electrostatic potential (MEP) map, we can assess the distribution of charges on the exterior surfaces of molecules, thereby providing information regarding how molecules interact with GHB. These potentials show that certain areas are positively charged due to a lack of electrons (thus creating an electropositive site), while other portions are negatively charged due to an excess of electrons (thus creating an electronegative site). Thus, MEP contours provide a means for predicting where molecular reactions or binding will occur (Fig. 4)^44,45^.

The MEP surface of pristine C_60_ is predominantly uniform throughout the fullerene cage due to its high symmetry and delocalized π-electron system. It has weakly negative electrostatic potentials, indicating that there are no strongly localized electrophilic or nucleophilic centers on its surface. In contrast, when heteroatoms, such as B or Zn, are added into the fullerenes C_59_, it changes the MEP surface by creating new regions of localized positive electrostatic potentials. For example, in BC_59_, the localized positive electrostatic potential surrounding the boron atom is attributed to its electron deficiency as compared with that of carbon atoms; therefore, we can conclude that this is a potential electrophilic center. ZnC_59_ has a similar localized positive electrostatic potential at the zinc site, indicating that this site has greater electron deficiency and increased electrophilic character as compared to C_60_ or BC_59_.

In contrast to C_60_ and BC_59_, GHB exhibits a far greater degree of MEP anisotropy than these molecules; the highest concentration of negative electrostatic potential occurs at the oxygen atoms where their lone pair of electrons are located; whereas, the majority of the positive electrostatic potentials occur around the hydrogen atoms and carbon centers which have no electrons. This distribution suggests that GHB can act as an electron donor in its interactions with an electrophilic surface. According to these results, it seems that the most likely interaction would be between the electron-deficient B or Zn atoms and the electron-rich oxygen atom of GHB. Based on these predictions, the desired complexes were designed and their optimal geometries were optimized (see Fig. 4).

Upon formation of the sensor@GHB complexes, a clear redistribution of electrostatic potential is observed (See Fig. 4, bottom row). In the C_60_@GHB system, only minor polarization of the MEP surface occurs, indicating relatively weak interaction dominated by noncovalent forces. In contrast, the BC_59_@GHB complex exhibits enhanced electrostatic complementarity, where the negative electrostatic potential regions of GHB are oriented toward the positively charged boron site, signifying stronger donor–acceptor interactions. The most significant MEP redistribution is observed for the ZnC_59_@GHB complex, in which the negative electrostatic potential regions of GHB are strongly aligned toward the highly positive Zn center. This pronounced electrostatic attraction suggests substantial charge transfer and indicates that Zn doping most effectively enhances the interaction between the fullerene surface and GHB. In general, based on the results of MEP analysis, heteroatom doping creates localized electrophilic sites on the fullerene cage, which is expected to enhance the electrostatic interactions with GHB and improve the sensing capability of the doped fullerene systems. This conclusion is analyzed in more detail in the following sections.

**Fig. 5:**
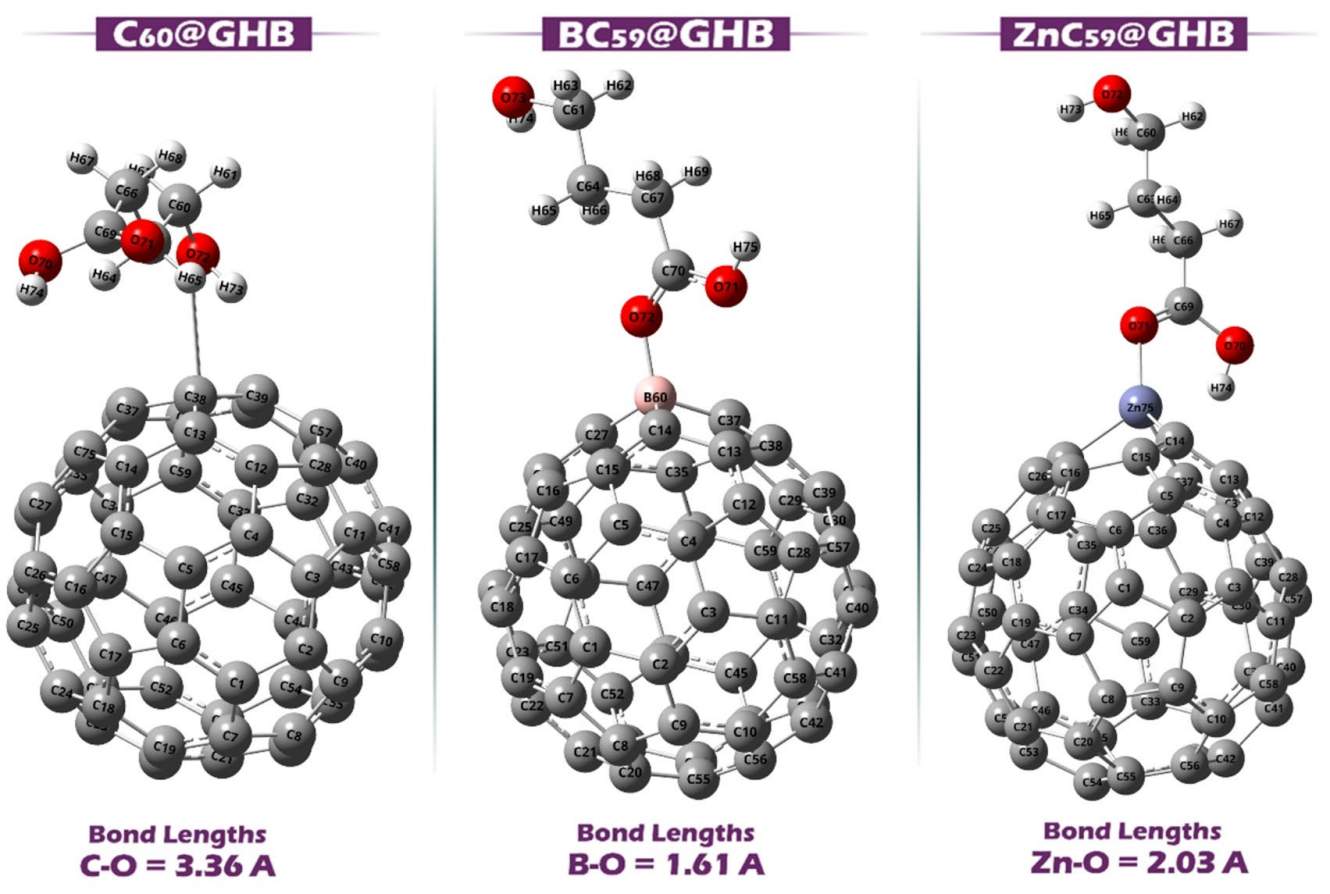
Optimized structure of Sensor@GHB complexes.

Figure 5 shows the optimized geometry of the C_60_@GHB, BC_59_@GHB, and ZnC_59_@GHB complexes. The bond lengths between the atoms involved in these complexes provide important insights into the strength and nature of the interactions between the sensor molecules (C_60_, BC_59_, and ZnC_59_) and GHB. In the C_60_@GHB complex, the bond length between the carbon atom in C_60_ and the oxygen atom in GHB is 3.36 Å. This relatively longer bond length suggests a weaker interaction between the two molecules. This may be because, in C_60_, the uniform electron distribution does not concentrate charge at a specific site, leading to a weaker bonding interaction with GHB’s oxygen atom. For the BC_59_@GHB complex, the bond length between boron (B) and oxygen (O) is 1.61 Å. This shorter bond length indicates a stronger interaction between the boron atom and the oxygen atom of GHB.

For the molecular complex ZnC_59_@GHB, the bond distance of Zn-O is 2.03 Å, which is intermediate between the shorter length of the bond for BC_59_@GHB and the longer length of the bond for C_60_@GHB. In comparison to boron (B), which is also electron deficient, Zn has an increased atomic radius and therefore has a longer bond length as well. Nevertheless, the interaction between the Zn atom and the GHB’s O atom is thought to be quite strong. Despite this, the interaction between the Zn atom and the oxygen atom in GHB remains relatively strong. According to the results, BC_59_ and ZnC_59_ are expected to form stronger interactions with GHB (this prediction will be examined in detail in the following sections by calculating adsorption energies).

**Fig. 6:**
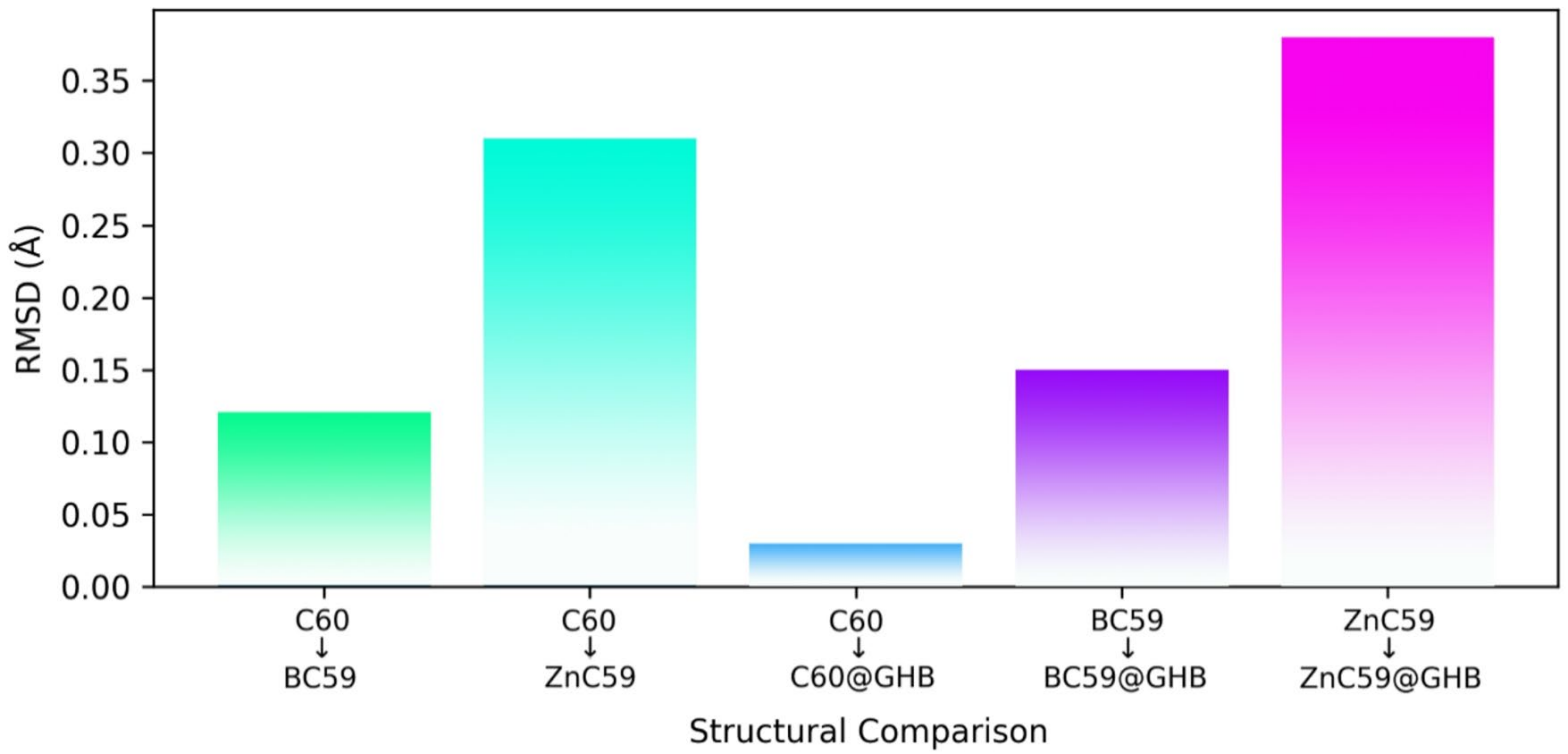
Root-mean-square deviation (RMSD) values for pristine and doped fullerene structures before and after GHB adsorption.

To evaluate quantitatively how doping would induce structural variability in conjunction with GHB adsorption, root-mean-square deviations (RMSD) from the pristine C_60_ for BC_59_ and ZnC_59_ have been calculated (see Fig. 6). The RMSD calculations were generated through the optimal alignment of atomic positions (taking only into account the fullerene framework) for both BC_59_ and ZnC_59_. The calculated RMSD of BC_59_ was modest and reflected localized distortion near the boron atom, whereas the calculated RMSD of ZnC_59_ was quite large, indicating significant deformation of the fullerene framework. Upon GHB adsorption on C_60_, there was little distortion; upon adsorption of GHB on BC_59_ there was moderate distortion; and upon GHB binding to the ZnC_59_ cage there was severe reorganization of the ZnC_59_ cage, thus indicating that the level of strain is increasing from C_60_ < BC_59_ < ZnC_59_.

### Reactivity and charge transfer parameters

As sensor materials are designed, the most important reactivity parameters (energy gap, chemical hardness, chemical softness, and chemical potential) help determine how a given sensor material will interact with its environment. A lower energy gap means that a sensor material is more chemically reactive. Chemical hardness is a measure of the resistance to deformation of the electron cloud surrounding a molecule; chemical softness is how easily a molecule will either donate electrons or accept electrons. Chemical potential is the tendency of a molecule to gain or to lose electrons, which is a critical factor in the sensing process. Charge transfer parameters, including _max_imum possible charge transfer (ΔN_max_) and charge transfer based on electrophilicity (ECT), evaluate a material’s ability to transfer electrons^46^. Thus, sensor materials tend to be those with lower hardness, higher softness, and better charge transfer characteristics, since they respond more readily to changes in their environment^46^. Therefore, a computational analysis of each of these parameters was performed (Table 2).

**Table 2:** Energy values ​​of orbitals HOMO (eV) and LUMO (eV) as well as HLG (eV), η (eV), S (eV^− 1^), µ (eV), ∆N_max_ and ECT for each of the conformers studied in this work.

Structure	LUMO	HOMO	HLG	η	µ	S	$\:\boldsymbol{\varphi\:}$	∆*N*_max_	ECT
C_60_	−3.58	−5.25	1.67 Theory	0.83	−4.41	1.20	2.625	5.28	------
			1.85 Exp^47^						
			1.86 Exp^48^						
			1.50 Exp^49^						
C_60_@GHB	−3.61	−5.27	1.66	0.83	−4.44	1.20	4.44	5.34	−0.06
BC_59_	−3.58	−4.93	1.35	0.67	−4.25	1.49	2.46	6.30	------
BC_59_@GHB	−3.34	−4.32	0.98	0.49	−3.83	2.04	3.83	7.81	−0.95
ZnC_59_	−4.09	−4.45	0.36	0.18	−4.27	5.55	4.27	23.72	------
ZnC_59_@GHB	−4.13	−4.52	0.39	0.19	−4.32	5.26	4.32	22.17	1.54

The data in Table 3 provides an analysis of the electronic properties of C_60_, BC_59_, and ZnC_59_, both before and after doping, and in the presence or absence of GHB.

**Fig. 7:**
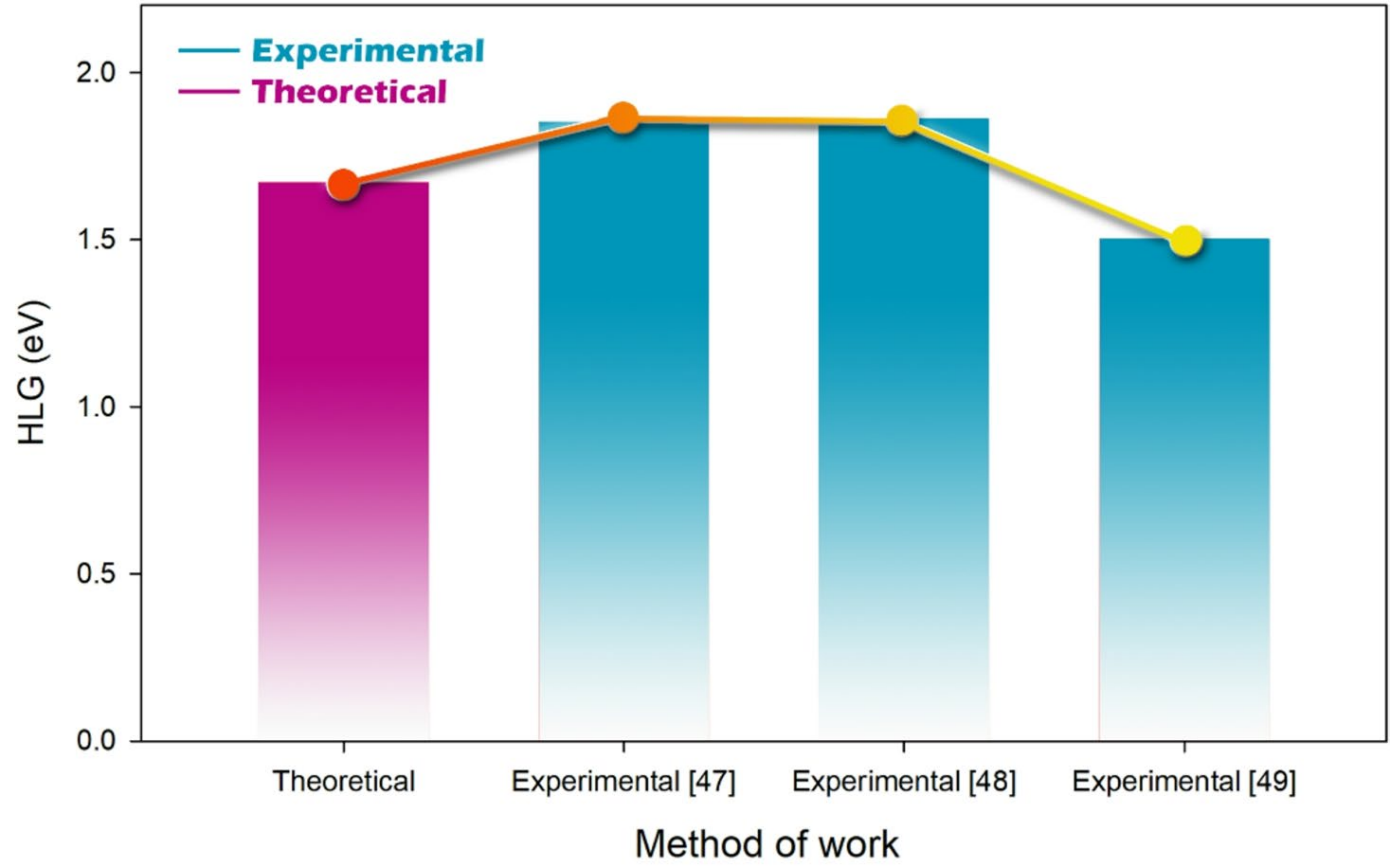
Qualitative comparison of the HLG obtained from the theoretical method (in this work) with experimental data in other papers (For Pristine C_60_).

For C_60_, the HOMO is at −5.25 eV and the LUMO at −3.58 eV, giving a HOMO-LUMO gap of 1.67 eV, indicating moderate reactivity (Fig. 7). The energy gap values calculated for C_60_ in this work agree well with experimental values reported in several studies, strengthening the validity of the computational approach used to investigate C_60_’s electronic properties and interactions. For instance, Rabenau et al. reported an energy gap of 1.85 eV for C_60_, determined from temperature-dependent microwave conductivity measurements^47^. This value is very close to our calculated energy gap, validating our computational method. Similarly, Kremer et al. conducted high-temperature conductivity studies on single-crystal C_60_ and found an energy gap of 1.86 eV, which also overlaps well with our result^48^. Additionally, Oshiyama et al. reported a slightly smaller energy gap of 1.50 eV for C_60_, based on their electronic-structure studies of fullerides^49^. While this value is somewhat lower than our calculated value, it is still within a reasonable range, reflecting the variability that can arise from different experimental methods and conditions (Fig. 7).

The chemical hardness (η) is 0.83 eV, and the chemical softness (S) is 0.59 eV, suggesting that C_60_ is relatively stable but still moderately reactive. The chemical potential (µ) is −4.41 eV, and the ΔN_max_ is 5.28, reflecting a moderate electron donation potential. When GHB is introduced (C_60_@GHB), the HOMO and LUMO energies shift slightly, resulting in a marginal decrease in the HOMO-LUMO gap to 1.66 eV. Other parameters such as η, µ, and S show minimal changes, but the ΔN_max_ increases slightly to 5.34. The most notable change is in the ECT, which shifts to −0.06, indicating that C_60_ becomes a mild electron acceptor in the presence of GHB. This suggests that GHB slightly alters C_60_’s electronic properties, making it more inclined to accept electrons.

For BC_59_, the HOMO is at −4.93 eV and the LUMO at −3.58 eV, resulting in a smaller HOMO-LUMO gap of 1.35 eV, indicating higher reactivity compared to C_60_. The chemical hardness (η) is lower (0.67 eV), and the chemical softness (S) is higher (0.74 eV), which suggests that BC_59_ is more reactive and more likely to donate electrons. The chemical potential (µ) is −4.25 eV, and the ΔN_max_ is 6.30, which reflects a higher electron donation potential than C_60_. When GHB is introduced (BC_59_@GHB), the HOMO and LUMO shift to −4.32 eV and − 3.34 eV, respectively, resulting in a significant decrease in the HOMO-LUMO gap to 0.98 eV. This indicates that BC_59_ becomes even more reactive with GHB, and the chemical hardness decreases to 0.49 eV, while the chemical softness increases to 1.02 eV. The chemical potential becomes more negative (−3.83 eV), further enhancing the electron donation ability of BC_59_. The ΔN_max_ increases to 7.81, indicating that BC_59_ can transfer more electrons in the presence of GHB. The ECT value drops to −0.95, indicating that BC_59_ behaves as a strong electron acceptor when GHB is present.

For ZnC_59_, the HOMO is at −4.45 eV and the LUMO at −4.09 eV, leading to a very small HOMO-LUMO gap of 0.36 eV, indicating high reactivity. The chemical hardness (η) is very low (0.18 eV), and the chemical softness (S) is very high (2.77 eV), suggesting that ZnC_59_ is highly reactive and easily accepts or donates electrons. The chemical potential (µ) is −4.27 eV, and the ΔN_max_ is very high at 23.72, indicating a strong electron transfer potential. When GHB is added (ZnC_59_@GHB), the HOMO shifts to −4.52 eV and the LUMO to −4.13 eV, resulting in a small increase in the HOMO-LUMO gap to 0.39 eV. The chemical hardness (η) and chemical softness (S) show minimal changes, with η increasing slightly to 0.19 eV and S decreasing slightly to 2.56 eV. The chemical potential becomes more negative at −4.32 eV, while the ΔN_max_ decreases slightly to 22.17, indicating a slight reduction in electron transfer potential. The ECT value becomes positive (1.54), suggesting that ZnC_59_ behaves as an electron donor in the presence of GHB.

**Fig. 8:**
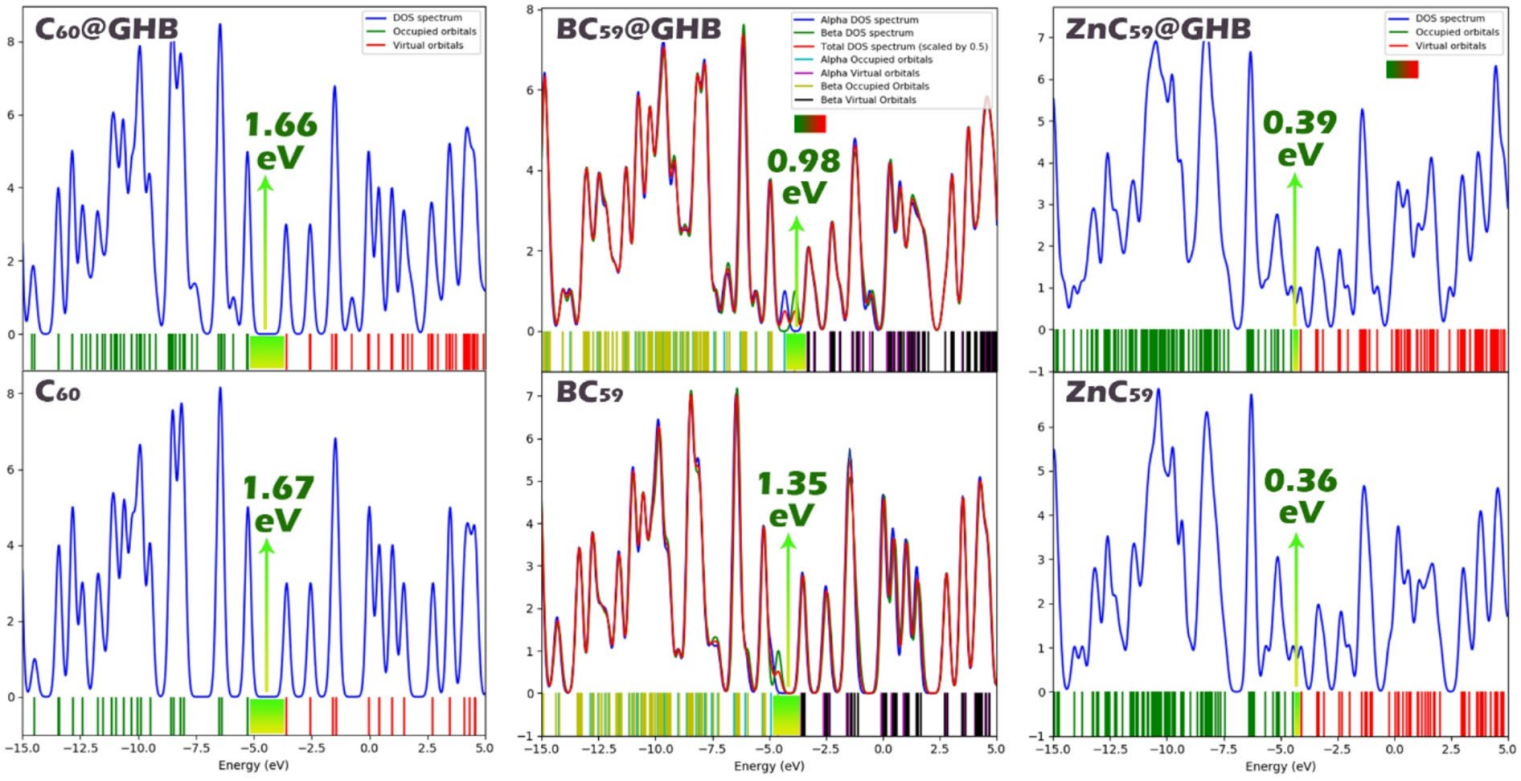
DOS spectra for each of the sensors in isolation and also their complexes with GHB.

The Density of States (DOS) plot provide an in-depth view of how sensor molecules interact with the GHB molecule. Analysis of the DOS plots shows that GHB adsorption causes a slight redistribution of electronic states near the Fermi level (this point was further investigated using PDOS and TDOS contours). This point is clearly seen for C_60_ near the Fermi level, confirming that their interaction is dominated by weak van der Waals forces. On the other hand, Boron doping results in new electronic states appearing very close to the Fermi level in BC_59_ and then after adsorption of GHB the density of those states grows. The dispersion of states directly leads to the decreased energy difference between the HOMO and LUMO, plus enables higher conductivity. With respect to the DOS profile of the ZnC_59_, the DOS density is very high at the Fermi level, which indicates a metal-like character prior to the adsorption of GHB. The minor change in the DOS for ZnC_59_ suggests that there is a minimal alteration of the ZnC_59_ conductivity as a result of strong GHB binding (Fig. 8). Collectively, this study indicates that the results obtained from the DOS diagrams of each sensor both with and without GHB correlated well with data in Table 2.

**Fig. 9:**
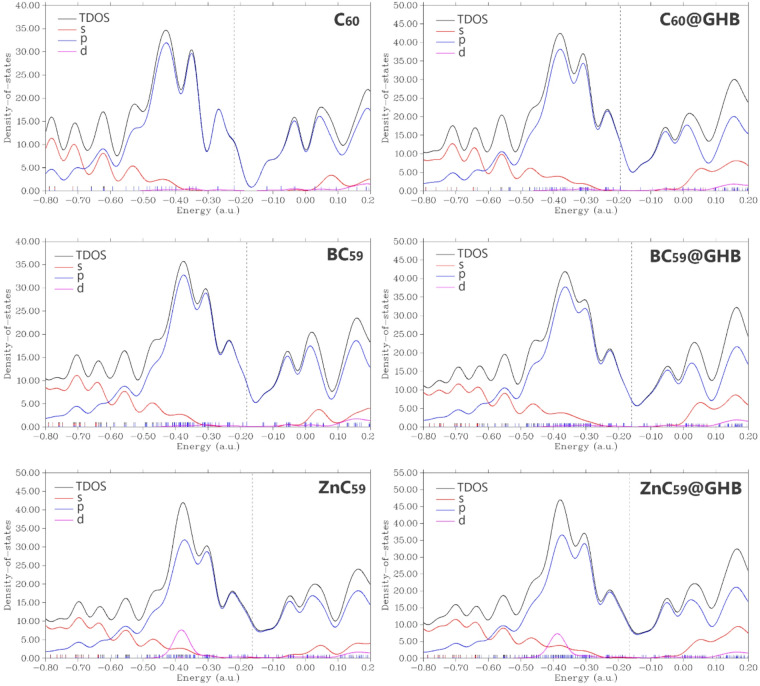
Total and projected density of states (TDOS/PDOS) for pristine and doped C_60_ (BC_59_, ZnC_59_) before and after interaction with GHB, with the Fermi level indicated by the dashed line.

Figure 9 presents total and projected densities of states (total density of states (TDOS) and partial density of states (PDOS)) for pristine C_60_, BC_59_, and ZnC_59_, each considered in isolation and when encapsulated or supported by GHB^51^. The vertical dashed line marks the Fermi level, so features to the left correspond to occupied states and those to the right to unoccupied states.

For pristine C_60_, the electronic structure is dominated by p-states over the entire energy range, reflecting the sp^2^ hybridized carbon network. The valence band shows several sharp peaks between roughly − 0.6 and − 0.3 a.u., while the conduction region begins just above the Fermi level with relatively low DOS, consistent with a molecular semiconductor featuring a clear HOMO-LUMO gap. The s and d contributions are negligible, indicating minimal orbital mixing beyond carbon p-character. Upon interaction with GHB (C_60_@GHB), the overall shape of the DOS is preserved, but the peaks broaden and the DOS near the Fermi level slightly increases. This indicates weak but non-negligible interaction between C_60_ and the host, leading to mild hybridization and partial charge redistribution without destroying the intrinsic electronic identity of C_60_.

In BC_59_, boron substitution introduces noticeable changes in the valence region. Additional states appear closer to the Fermi level, and the p-derived DOS is enhanced near the top of the valence band. This reflects the electron-deficient nature of boron, which acts as a p-type dopant and effectively shifts spectral weight toward higher energies. The HLG is reduced compared with pristine C_60_, suggesting improved electronic conductivity. When BC_59_ is combined with GHB, these effects are amplified: peak broadening increases and the DOS around the Fermi level rises further, indicating stronger electronic coupling and enhanced charge-transfer interactions relative to C_60_@GHB.

ZnC_59_ shows the most pronounced modification of the electronic structure. In addition to dominant carbon p-states, clear d-state contributions emerge in the valence band around − 0.45 to −0.35 a.u., originating from Zn d orbitals. These d states hybridize with carbon p orbitals, producing higher TDOS peaks and a further reduction of the gap. The presence of Zn thus introduces localized metal-derived states that significantly alter the electronic landscape. Encapsulation or support by GHB (ZnC_59_@GHB) enhances these trends: the TDOS increases across both valence and conduction regions, peak structures broaden substantially, and the DOS at the Fermi level is the highest among all systems considered. It is predicted that this behavior can lead to the strongest host-guest interaction and the highest degree of electronic delocalization and charge transfer, which will be studied in detail in the following sections.

**Fig. 10:**
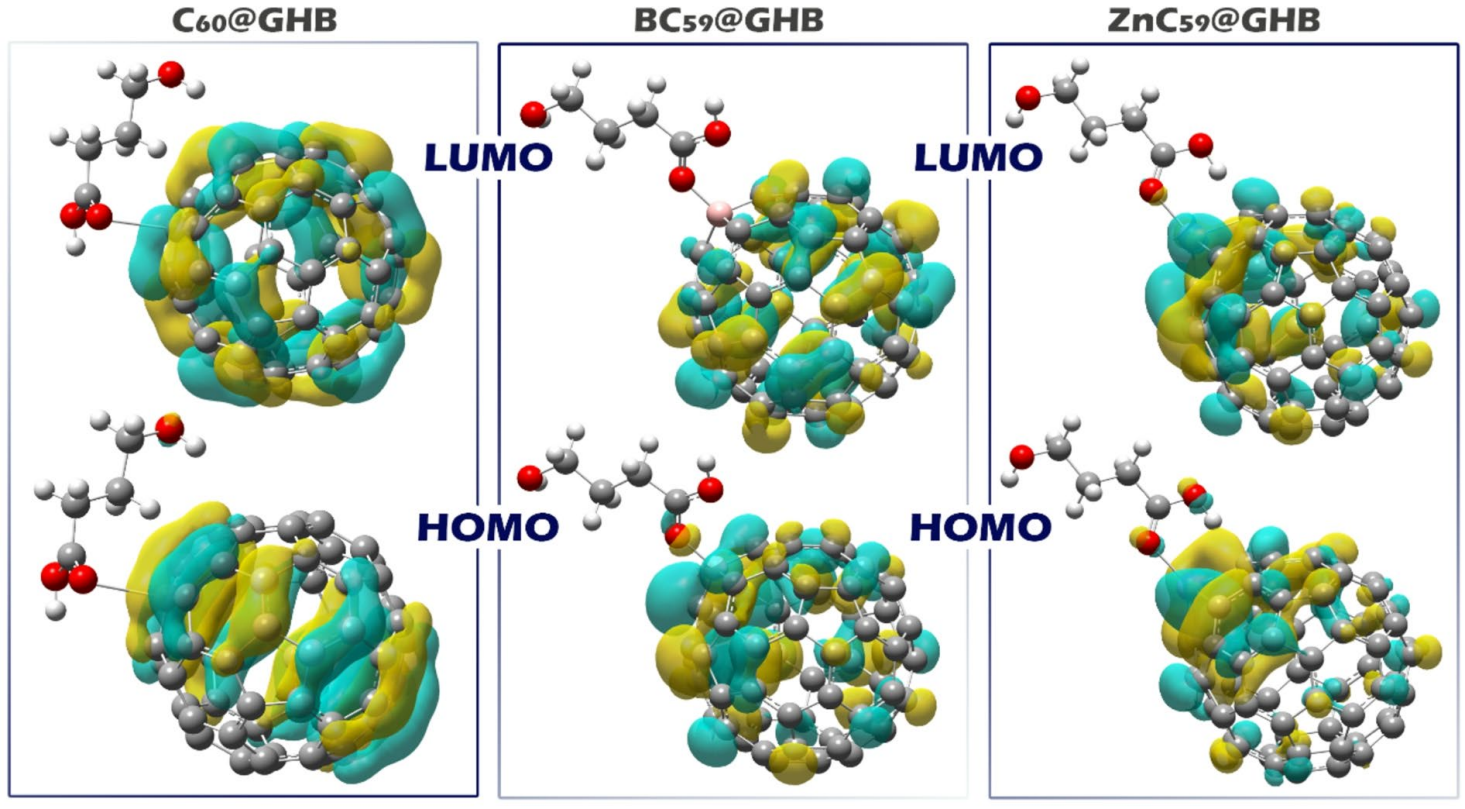
Spatial distributions and phase patterns of the HOMOs (bottom row) and LUMOs (top row) for C_60_@GHB, BC_59_@GHB, and ZnC_59_@GHB. The blue and cyan colors indicate positive and negative phases of the electron wave function, respectively.

The distribution of HOMO and LUMO orbitals in a complex is a key factor in determining how the molecule behaves in chemical reactions, its stability, and its interactions with other species. In sensors, knowing the location of the HOMO and LUMO helps in designing materials that can selectively interact with target molecules, optimizing sensitivity and selectivity. For example, if the HOMO is located in a particular region of the molecule, it indicates that the complex is likely to donate electrons to neighboring molecules. Conversely, if the LUMO is localized, the complex may have a greater tendency to accept electrons from donor molecules. In all the designed complexes, both the HOMO and LUMO orbitals are located on the sensor^52^. This configuration ensures that the sensor can efficiently engage in both electron donation (via the HOMO) and electron acceptance (via the LUMO), making it highly responsive to changes in its environment (Fig. 10).

### Dipole moment and Polarizability

Studying the dipole moment and polarizability of a molecule is important because of its direct effect on solubility and ability to generate electrical signals. Molecules with a higher dipole moment tend to be more soluble in polar solvents due to favorable interactions between the solvent and the molecule. On the other hand, high polarizability enhances a molecule’s response to electric fields, making it more efficient in generating electrical signals in sensors or electronic devices^53,54^. Each of these parameters was calculated, and the results were reported in Table 3.

**Table 3:** Values of Dipole moments ​​and polarizability for each designed structure.

Structure	Dipole moments (Debye)	Polarizability (a.u.)
C_60_	0.00	483.75
C_60_@GHB	1.25	546.54
BC_59_	1.51	501.49
BC_59_@GHB	16.23	574.31
ZnC_59_	6.24	530.98
ZnC_59_@GHB	6.14	604.05

**Fig. 11:**
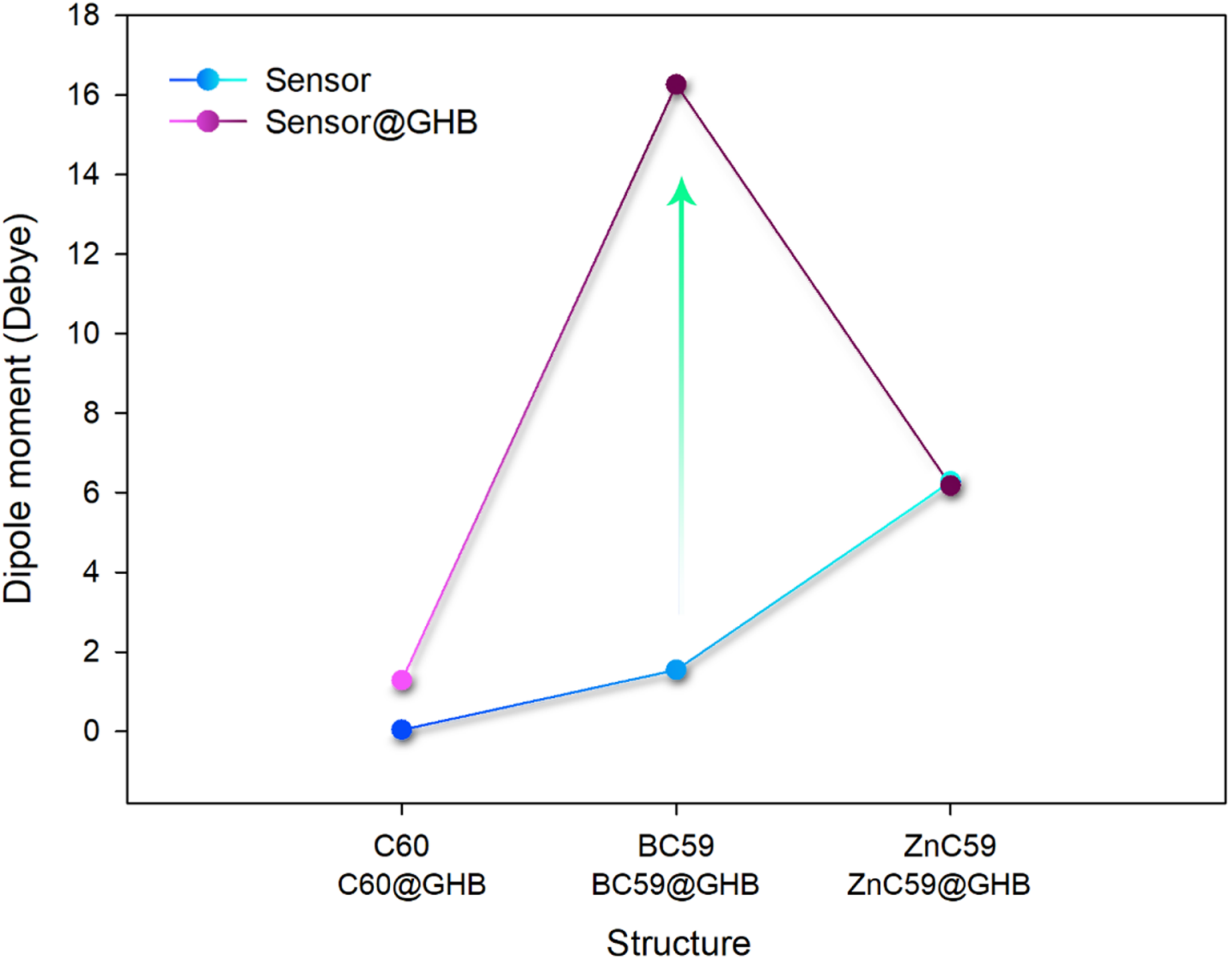
Qualitative representation of the dipole moment changes for each of the designed sensors in the presence/absence of GHB.

The dipole moment of a molecule is a key factor in determining its solubility, especially in polar solvents. A higher dipole moment indicates a greater polarity, which generally results in better solubility in polar solvents. C_60_ has a dipole moment of 0.00 Debye, indicating it is nonpolar and therefore poorly soluble in polar solvents. However, when doped with GHB (C_60_@GHB), the dipole moment increases to 1.25 Debye, suggesting that the molecule becomes more polar and, as a result, more soluble in polar solvents. BC_59_ has a dipole moment of 1.51 Debye, indicating moderate polarity and suggesting it has some solubility in polar solvents. With GHB doping, the dipole moment increases dramatically to 16.23 Debye, making BC_59_@GHB highly polar and significantly more soluble in polar solvents. ZnC_59_, with a dipole moment of 6.24 Debye, has moderate polarity, offering good solubility in polar solvents. The dipole moment decreases slightly to 6.14 Debye in ZnC_59_@GHB, suggesting a small reduction in solubility, but it remains highly soluble compared to C_60_ (Fig. 11).

The higher the polarizability, the better the material is at generating electrical signals. C_60_ has a polarizability of 483.75 a.u., which is relatively low, suggesting modest electrical conductivity. With GHB doping (C_60_@GHB), the polarizability increases slightly to 546.54 a.u., indicating a small improvement in electrical conductivity. BC_59_ has a polarizability of 501.49 a.u., which is slightly higher than C_60_, indicating better potential for electrical conductivity. With GHB doping (BC_59_@GHB), the polarizability increases to 574.31 a.u., resulting in a noticeable improvement in electrical conductivity. ZnC_59_ has a polarizability of 530.98 a.u., which is higher than both C_60_ and BC_59_, suggesting good electrical conductivity. When doped with GHB (ZnC_59_@GHB), the polarizability increases to _60_4.05 a.u., marking the highest value among all the structures. This indicates that ZnC_59_@GHB would exhibit the best electrical conductivity. According to the reported results, BC_59_ and ZnC_59_ show higher polarizability values ​​when in the presence of GHB than in the isolated state. Therefore, both structures are expected to produce better electrical signals in the presence of GHB.

#### Sensor application

The adsorption energy, recovery time, and electrical conductivity are critical parameters for evaluating the ability of a structure to function as an effective sensor. The adsorption energy indicates the strength of the binding in the complex. The recovery time is the time it takes for the sensor to return to its ground state after detecting a substance, and the electrical conductivity determines the ability of the sensor to generate an electrical signal in response to the absorption of the analyte^55,56^. Each of these parameters was carefully calculated, and the results are reported in Table 4.

**Table 4:** Adsorption energy (Eads), electrical conductivity ($\:\boldsymbol{\sigma\:}$) and recovery time ($\:\boldsymbol{\tau\:}$) values ​​in each of the studied structures.

Structure	E_ads_ (kcal.mol^− 1^)	$\:\boldsymbol{\tau\:}$ (s)	Log 10 ($\:\boldsymbol{\tau\:}$)	($\:\boldsymbol{\sigma\:}$) (S/m)
C_60_	**-----**	**-----**	**-----**	2.20 × 10^9^
C_60_@GHB	−14.03	1.94 × 10^− 2^	−1.71	2.21 × 10^9^
BC_59_	**-----**	**-----**	**-----**	2.35 × 10^9^
BC_59_@GHB	−23.11	1.51 × 10^5^	5.18	2.53 × 10^9^
ZnC_59_	**-----**	**-----**	**-----**	2.87 × 10^9^
ZnC_59_@GHB	−26.91	4.27 × 10^7^	7.63	2.85 × 10^9^

The calculated adsorption energies reveal a clear trend in the interaction strength between GHB and the studied nanostructures. C_60_@GHB exhibits a relatively weak interaction with an adsorption energy of −14.03 kcal.mol^− 1^, suggesting predominantly physisorption. In contrast, BC_59_@GHB and ZnC_59_@GHB show significantly more negative adsorption energies of −23.11 and − 26.91 kcal.mol^− 1^, respectively, indicating much stronger binding. This strong interaction is further supported by the recovery time analysis.

**Fig. 12:**
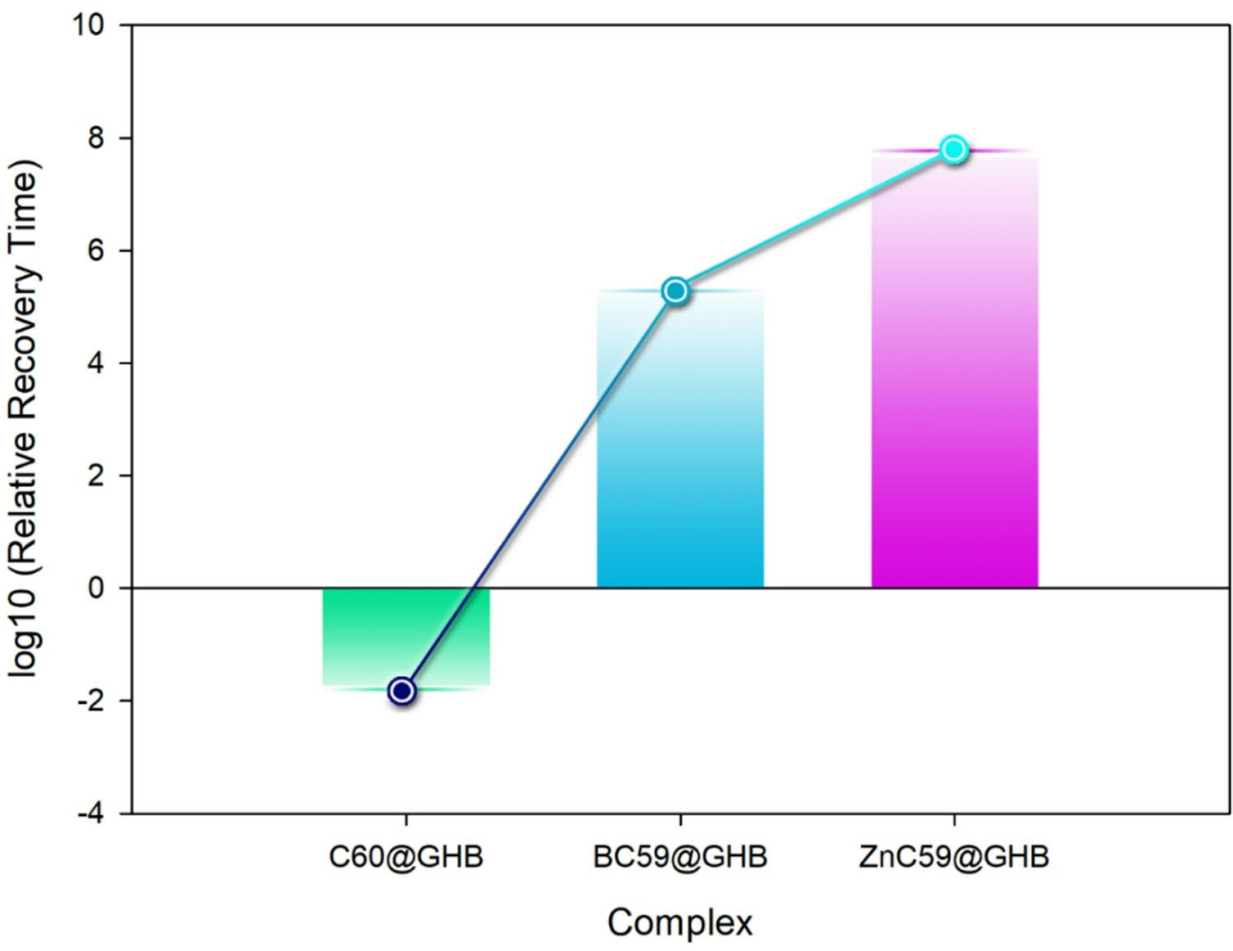
Logarithmic (log10) comparison of recovery time (τ) for C_60_@GHB, BC_59_@GHB, and ZnC_59_@GHB systems, illustrating relative desorption trends derived from computational analysis. The logarithmic representation is employed to emphasize qualitative trends rather than absolute quantitative values.

The very short recovery time of C_60_@GHB (1.94 × 10^− 2^ s) indicates rapid desorption, which is consistent with its weak adsorption energy and limits its usefulness for effective GHB capture. In contrast, BC_59_@GHB and ZnC_59_@GHB exhibit extremely long recovery times (1.51 × 10^5^ s and 4.27 × 10^7^ s, respectively). The exceptionally long τ value for ZnC_59_@GHB confirms that GHB is strongly retained on the surface, making desorption kinetically unfavorable. Such behavior is highly desirable for adsorption and removal applications, where stable and irreversible binding is required. Figure 8 shows a logarithmic comparison of recovery time trends for the investigated systems upon GHB adsorption. Among the complexes, ZnC_59_@GHB exhibits the longest recovery trend, indicating comparatively stronger interaction between the adsorbate and the doped surface. BC_59_@GHB displays an intermediate recovery behavior, whereas C_60_@GHB shows the shortest recovery trend, suggesting relatively faster desorption characteristics. It should be emphasized that these recovery times are obtained from computational calculations and are intended to represent qualitative, relative trends rather than exact experimental values. Experimentally, desorption kinetics may be significantly accelerated through external regeneration strategies (Fig. 12).

Electrical conductivity changes upon analyte adsorption are a decisive factor for electrochemical sensing. C_60_ shows negligible conductivity variation upon GHB adsorption, indicating poor sensitivity. ZnC_59_ also exhibits only a slight decrease in conductivity after adsorption, despite its strong binding capability, which limits its effectiveness as a sensing material. In contrast, BC_59_ demonstrates a noticeable increase in electrical conductivity from 2.35 × 10^9^ to 2.53 × 10^9^ S.m^− 1^ upon GHB adsorption. This significant conductivity modulation suggests effective charge transfer between GHB and the BC_59_ framework, resulting in a clear electrical signal. The substantial conductivity change enables sensitive detection, while its long recovery time supports disposable sensor applications.

**Fig. 13:**
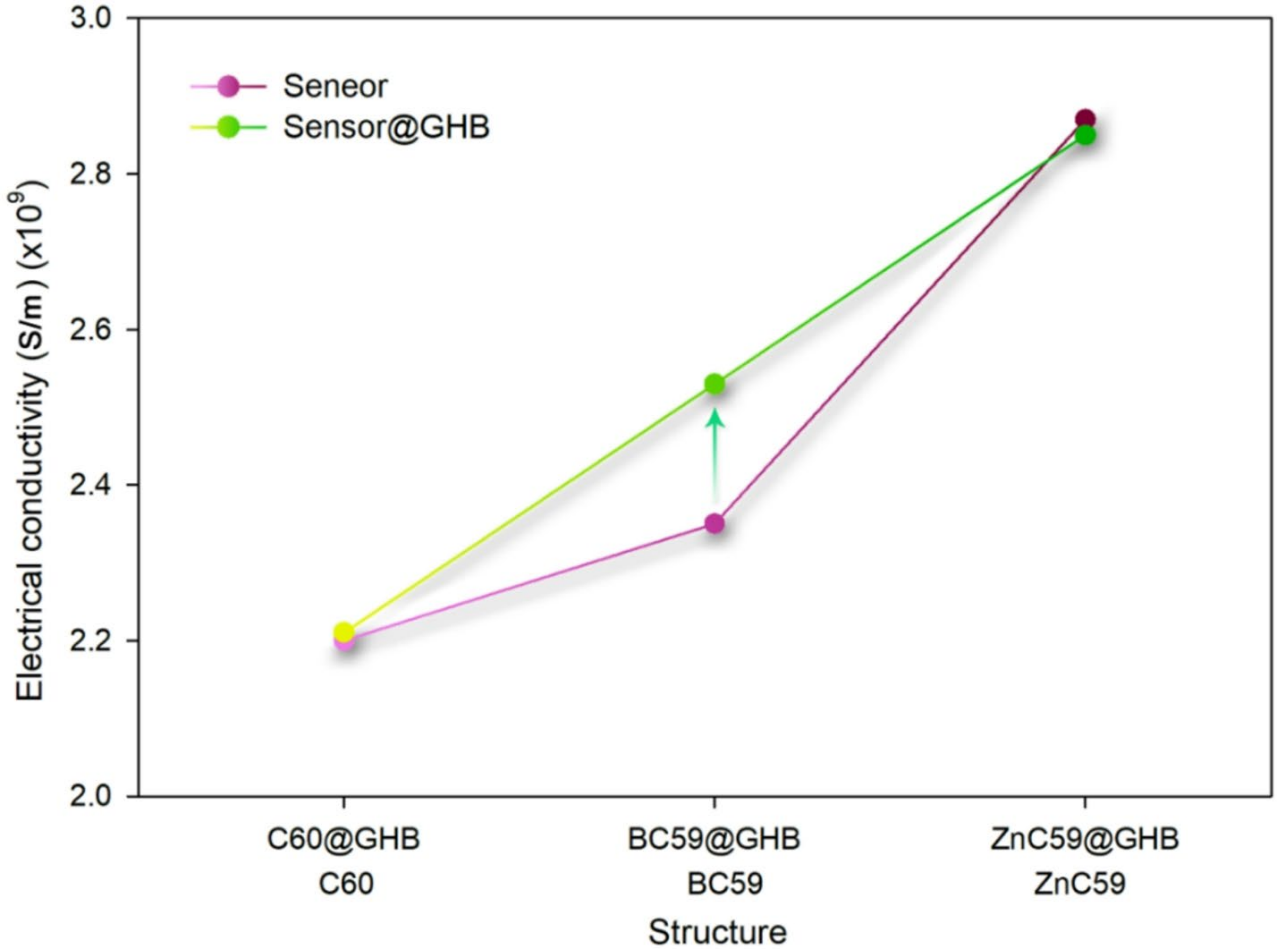
Investigating comparative electrical conductivity sensing behavior instead of absolute numerical predictions for each of the designed sensors in the presence/absence of GHB.

In Fig. 13, we discuss the qualitative behavior of electrical conductivity in a comparative manner to highlight the relative trends in the systems. It should be emphasized that since the present study is purely computational, this quantity is not intended to represent absolute experimental values, but rather is useful as a qualitative indicator for the relative evaluation of the sensing behavior. This table emphasizes that for BC_59_ after GHB adsorption, the change in electrical conductivity can be more significant than for other structures.

### UV Spectrum

**Fig. 14:**
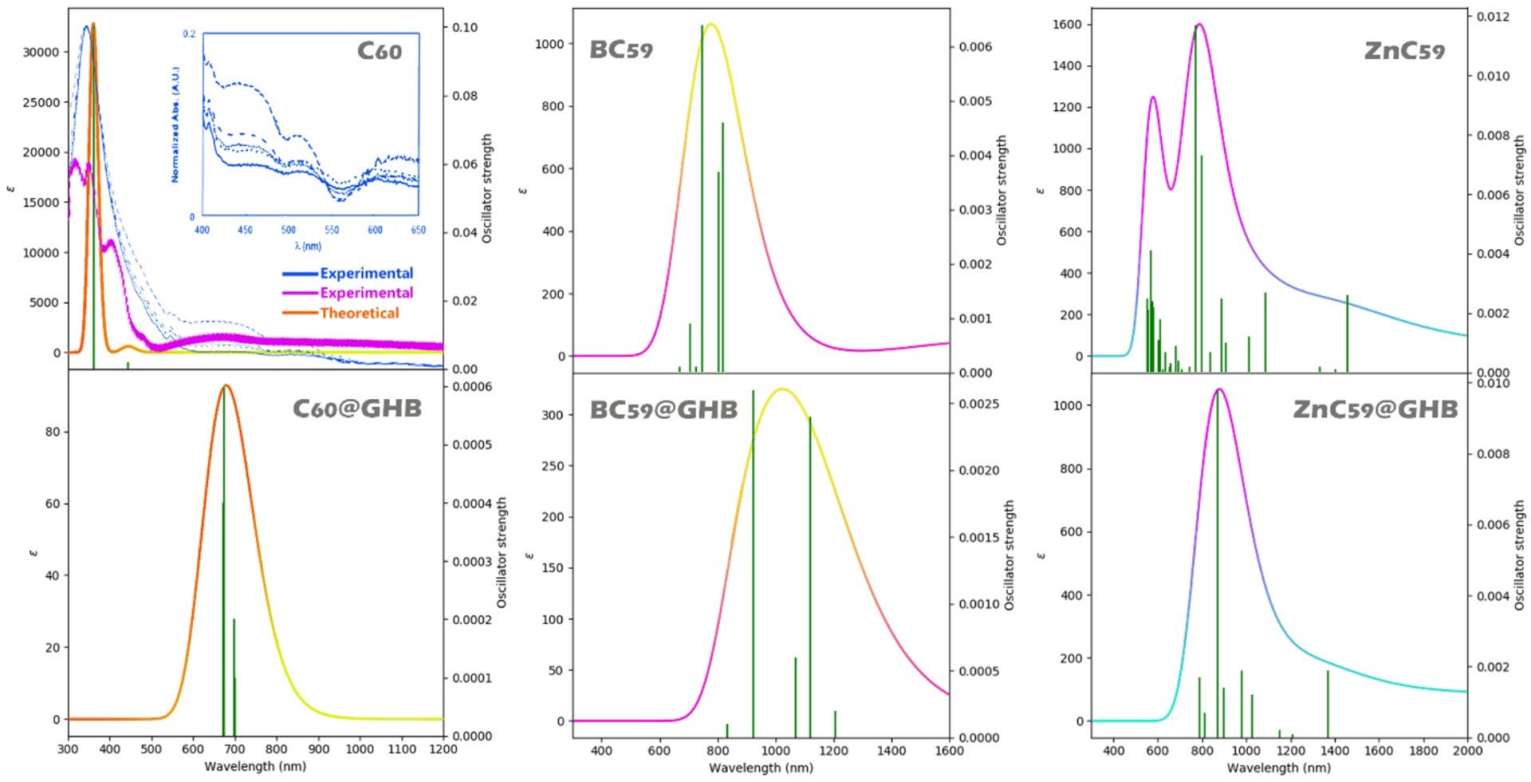
Visual representation of the λ_max_ calculated from the theoretical method (along with the experimental λ_max_ for C_60_) and the corresponding excitation energies (Eex) for each of them with and without GHB.

The colorimetric performance of a sensor can be evaluated by examining changes in the _max_imum absorption wavelength (λ_max_) and exciton energy (Eex). In general, a red shift in λ_max_ accompanied by a decrease in exciton energy indicates stronger electronic coupling, enhanced charge-transfer character, and improved light absorption in the visible to near-infrared region. Such features are favorable for colorimetric sensing because they increase the likelihood of an observable color change that can be detected by the naked eye or simple optical tools^57^. For this purpose, each of these parameters was computationally studied for the designed structures, and the results were reported in Table 5; Fig. 14.

**Table 5:** Theoretical absorption _max_imum wavelengths (along with experimental values ​​for C_60_) (λ_max_) and corresponding excitation energies (Eex) for pristine and doped C_60_ systems with and without GHB.

Structure	λ_max_ (nm)	Eex (eV)
C_60_	360 (Theoretical)	3.43
	335 (Experimental)^58^	-------
	345 (Experimental)^59^	-------
BC_59_	863	1.43
ZnC_59_	774	1.60
C_60_@GHB	674	1.83
BC_59_@GHB	924	1.34
ZnC_59_@GHB	870	1.42

C_60_ exhibits a λ_max_ of 360 nm in its unbound state, corresponding to absorption in the violet-blue region (≈ 2.78 eV). For pristine C_60_, the calculated absorption _max_imum at 3_60_ nm (Eex = 3.43 eV) shows good agreement with previously reported experimental values at 335 nm and 345 nm, confirming the reliability of the computational approach used in this work^58,59^. In the presence of GHB, the complex C_60_@GHB shows a marked redshift to 674 nm (1.83 eV), within the red region of the visible spectrum. This shift of 230 nm represents a dramatic change in the absorption profile, which would translate to a clear color transition (likely from yellowish (transmitting complementary to blue absorption) to bluish-green (transmitting complementary to red absorption)). Both absorption maxima reside within the visible range, ensuring that the color change is perceptible to the unaided eye.

In contrast, BC_59_ and ZnC_59_ display λ_max_ values in the infrared region both before and after GHB binding (BC_59_: 863 nm → 924 nm; ZnC_59_: 774 nm → 870 nm). Although these materials may exhibit weak visible coloring due to absorption tails extending into the red edge of the spectrum, their primary absorption bands lie outside human vision. Any color change upon GHB binding would be subtle and unreliable for visual detection, as the major shift occurs in the infrared, beyond the sensitivity of the human eye.

Based on comparative analysis of optical absorption data, C_60_ is proposed as an optimal candidate for a GHB colorimetric sensor whose absorption _max_imum shifts completely into the visible spectrum upon analyte binding, ensuring a detectable color change that can be observed without specialized equipment. The UV-visible spectra in Fig. 14 confirm the quantitative data in Table 5 and show that analyte binding causes significant λ_max_ shifts and exciton energy reduction. Among all the systems, C_60_@GHB shows the most suitable spectral changes, supporting its identification as the most promising colorimetric sensor due to its improved optical response and higher probability of visible color change.

To further distinguish between reversible sensing and permanent adsorption behavior, desorption energetics and temperature-dependent kinetics were considered. For non-dissociative adsorption, the desorption energy (Edes) can be approximated by the absolute value of the adsorption energy. Based on this approximation, pristine C_60_ exhibits a relatively low desorption barrier (≈ 14 kcal.mol^− 1^), which enables rapid thermal desorption at ambient temperature (298 K), consistent with its very short recovery time. This behavior confirms that GHB adsorption on pristine C_60_ is governed by weak physisorption, making it suitable for reversible sensing applications.

In contrast, the significantly higher desorption barriers for BC_59_ (≈ 23 kcal.mol^− 1^) and ZnC_59_ (≈ 27 kcal.mol^− 1^) lead to exponentially slower desorption kinetics at room temperature, as predicted by Arrhenius-type models. The resulting recovery times far exceed practical sensing cycles, particularly for ZnC_59_, indicating kinetically trapped adsorption. Consequently, BC_59_ and ZnC_59_ behave more like semi-permanent and permanent adsorbents, respectively, rather than reusable sensors. These kinetic trends are analyzed in more detail in the following sections by QTAIM, NCI/RDG, ELF, and LOL analyses.

### Natural bond orbitals (NBOs) analysis

NBO analysis is vital in designing Sensor@GHB complexes as it provides insights into the electronic interactions between the sensor and the drug molecules, helping optimize the sensor’s sensitivity, selectivity, and efficiency. A key parameter in this analysis is the second-order perturbation energy E^2^, which indicates the strength of orbital interactions (higher E^2^ values suggest a more efficient sensor with better drug-binding ability). NBO analysis also identifies various electronic transitions, including σ-σ*, π-π*, and LP(1)-π*, with π-π* transitions being the most important for sensor design. These transitions involve the movement of electrons between bonding and antibonding π orbitals, leading to significant changes in the sensor’s electronic properties, enhancing its ability to detect drugs effectively. Therefore, focusing on optimizing π-π* transitions can significantly improve the performance of sensor@GHB complexes^60,61^.

**Table 6:** Calculated values ​​of NBOs analysis for the studied complexes.

Complex	Donor (i)	Type	Aceptor (j)	Type	E^2^ kcal.mol^− 1^	E(j)-E(i) a.u.	F(i, j) a.u.
C_60_@GHB	C_1_-C_2_	$\:\sigma\:$	C_1_-C_6_	$\:{\sigma\:}^{*}$	2.32	1.08	0.045
	C_1_-C_6_	$\:\pi\:$	C_2_-C_3_	$\:{\pi\:}^{*}$	12.35	0.26	0.050
	O_71_	LP(1)	C_69_-O_70_	$\:{\pi\:}^{*}$	30.77	0.50	0.112
BC_59_@GHB	C_1_-C_2_	$\:\sigma\:$	C_3_-C_11_	$\:{\sigma\:}^{*}$	1.41	1.02	0.048
	C_17_-C_1_	$\:\pi\:$	C_7_-C_19_	$\:{\pi\:}^{*}$	7.33	0.25	0.055
	C_35_	LP(1)	C_27_-C_48_	$\:{\pi\:}^{*}$	44.37	0.12	0.102
ZnC_59_@GHB	C_1_-C_2_	$\:\sigma\:$	C_3_-C_11_	$\:{\sigma\:}^{*}$	3.22	0.94	0.049
	C_7_-C_19_	$\:\pi\:$	C_17_-C_18_	$\:{\pi\:}^{*}$	14.28	0.25	0.054
	C_26_	LP(1)	C_24_-C_25_	$\:{\pi\:}^{*}$	33.72	0.13	0.074

Based on the Natural Bond Orbital (NBO) analysis data provided, the three complexes (C_60_@GHB, BC_59_@GHB, and ZnC_59_@GHB) exhibit distinct electronic interaction profiles, revealing the fundamental differences in how GHB is stabilized on each surface (Table 6).

In the C_60_@GHB complex, the most significant stabilizing interaction is a strong lone pair donation. The lone pair (LP(1)) on the oxygen atom of GHB (O_71_) donates into the π* anti-bonding orbital of the C_69_-O_70_ bond within GHB itself, with a high second-order perturbation energy (E^2^) of 30.77 kcal.mol^− 1^. This indicates a pronounced intramolecular stabilization within the adsorbed GHB molecule. The primary stabilization from the C_60_ surface comes from a π(C_1_-C_6_) to π*(C_2_-C_3_) donation with an E^2^ of 12.35 kcal.mol^− 1^, suggesting a moderate π-π interaction between the fullerene and the molecule.

The BC_59_@GHB complex reveals a markedly different and more robust interaction mechanism. Here, the most powerful stabilization arises from the boron-doped carbon structure. A lone pair on a carbon atom (C_35_, likely adjacent to the electron-deficient boron) donates into a π* anti-bonding orbital (C_27_-C_48_) with a very high E^2^ value of 44.37 kcal.mol^− 1^. This is significantly stronger than the primary interaction in C_60_@GHB and points to a substantial charge transfer from the doped surface to the adsorbate framework. The π-to-π* interaction within the complex (C_17_-C_1_ to C_7_-C_19_) is weaker (E^2^ = 7.33 kcal.mol^− 1^) than in the C_60_ case, implying that the lone-pair donation is the dominant chemisorption force.

For the ZnC_59_@GHB complex, the NBO analysis shows a profile that is intermediate in strength but distinct in character. The key lone pair donation, from C_26_ to the π*(C_24_-C_25_) orbital, has an E^2^ value of 33.72 kcal.mol^− 1^. This is stronger than the lone pair interaction in C_60_@GHB but weaker than the exceptional one in BC_59_@GHB. Concurrently, the π-to-π* back-donation (C_7_-C_19_ to C_17_-C_18_) is the strongest among the three complexes at 14.28 kcal.mol^− 1^. This indicates a more balanced, dual-mechanism stabilization in ZnC_59_, with significant contributions from both surface-to-adsorbate donation and mutual π-system interactions.

In conclusion, the NBO analysis reveals a clear gradation in the nature and strength of the key stabilizing interactions: C_60_@GHB is primarily stabilized by intramolecular GHB interactions with a moderate surface contribution; BC_59_@GHB is dominated by an extremely strong charge transfer from the boron-doped surface; and ZnC_59_@GHB features a robust, balanced mix of strong surface donation and π-back-donation, correlating with its previously identified role as the strongest adsorbent.

### NCI/RDG Analysis

**Fig. 15:**
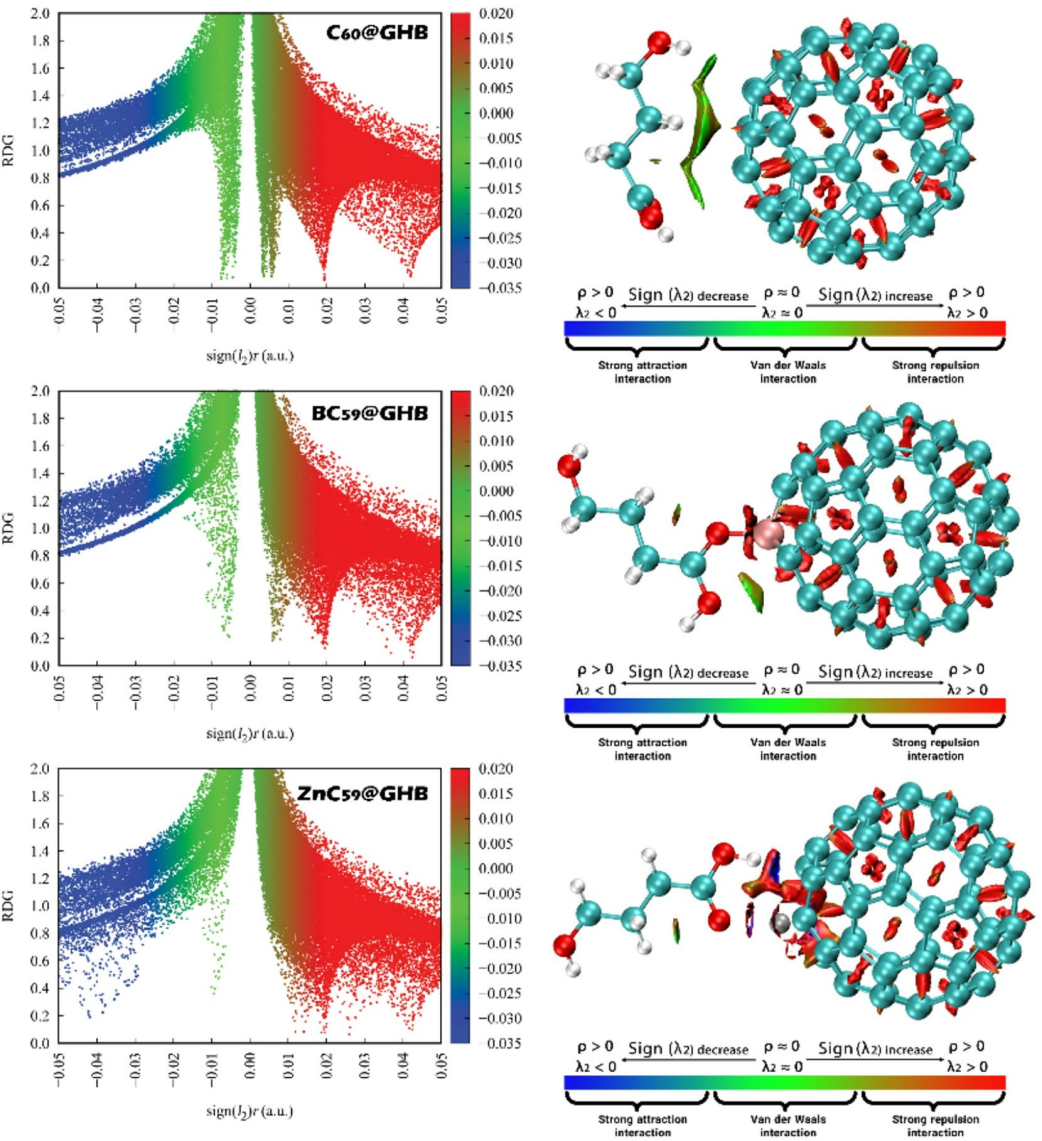
The NCI/RDG plot plots of the studied complexes, including C_60_@GHB, BC_59_@GHB and ZnC_59_@GHB.

Examining Non-Covalent Interaction (NCI) contours is crucial for studying intermolecular interactions because it provides a visual and quantitative representation of the spatial regions where non-covalent forces, such as hydrogen bonds, van der Waals interactions, and π-π stacking, play a significant role. These interactions are fundamental in determining the stability, reactivity, and functionality of molecular complexes, including drug-receptor binding, material properties, and molecular recognition processes. The NCI contour plots show the relationship between the sign(λ_2_), which indicates the nature of the non-covalent interaction (attractive or repulsive), and ρ (electron density), which is a measure of the local electron distribution around the molecules. In these plots, red regions indicate strong attractive interactions, green regions correspond to van der Waals interactions, and blue regions show strong repulsion^62,63^. Figure 15 shows the NCI/RDG contours for each of the designed complexes.

The NCI/RDG analysis reveals that noncovalent interactions between GHB and the fullerene-based hosts are predominantly weak and dispersion-driven, with their strength clearly modulated by heteroatom and metal doping. In the RDG versus sign(λ_2_)ρ plots, the main interaction features for all three systems appear at low RDG values and are concentrated around sign(λ_2_)ρ ≈ 0, which is characteristic of van der Waals interactions. For the C_60_@GHB system, the distribution is dominated by green regions near zero sign(λ_2_)ρ, indicating that the interaction between GHB and pristine C_60_ is mainly governed by weak dispersive forces, with negligible contributions from strong attractive interactions. The absence of pronounced negative sign(λ_2_)ρ spikes further confirms the lack of significant hydrogen bonding or electrostatic attraction, while the positive sign(λ_2_)ρ regions are largely associated with steric repulsion within the fullerene framework itself rather than destabilizing host–guest interactions.

Upon boron doping (BC_59_@GHB), the RDG scatter shows a noticeable increase in interaction density at lower RDG values, accompanied by a slight enhancement of features extending toward the negative sign(λ_2_)ρ region. This behavior suggests a modest strengthening of the host–guest interaction, which can be attributed to increased polarization and altered electronic distribution induced by B substitution. Nevertheless, the interaction remains largely noncovalent and vdW-dominated, as evidenced by the persistence of green regions as the primary feature in both the RDG plots and the NCI isosurfaces.

In the ZnC_59_@GHB system, the interaction becomes significantly stronger, as indicated by more pronounced blue-green spikes at low RDG values and the increased presence of negative sign(λ_2_)ρ features. These changes reflect enhanced attractive interactions arising from the incorporation of Zn, which introduces localized electrostatic and possible coordination effects that reinforce the binding between GHB and the fullerene cage. The corresponding NCI isosurfaces show more intense and localized blue and green regions at the host-guest interface, confirming the strengthened noncovalent interaction. Across all systems, red regions remain mainly confined to the fullerene framework, signifying steric repulsion intrinsic to the cage rather than unfavorable guest binding. Overall, the NCI/RDG results show a clear trend in the interaction strength C_60_@GHB < BC_59_@GHB < ZnC_59_@GHB, which is consistent with the reported adsorption energy.

### Localized orbital locator (LOL) and Electron Localization Function (ELF) analysis

The Localized Orbital Locator (LOL) maps provide a real-space description of electronic structure and host-guest interactions in the C_60_@GHB, BC_59_@GHB, and ZnC_59_@GHB complexes. LOL analysis highlights regions of orbital localization and electronic coupling, enabling a detailed understanding of adsorption mechanisms and interaction strength^64^. According to the obtained results, the LOL analysis shows a clear progression in the interaction strength: C_60_@GHB < BC_59_@GHB < ZnC_59_@GHB.

**Fig. 16:**
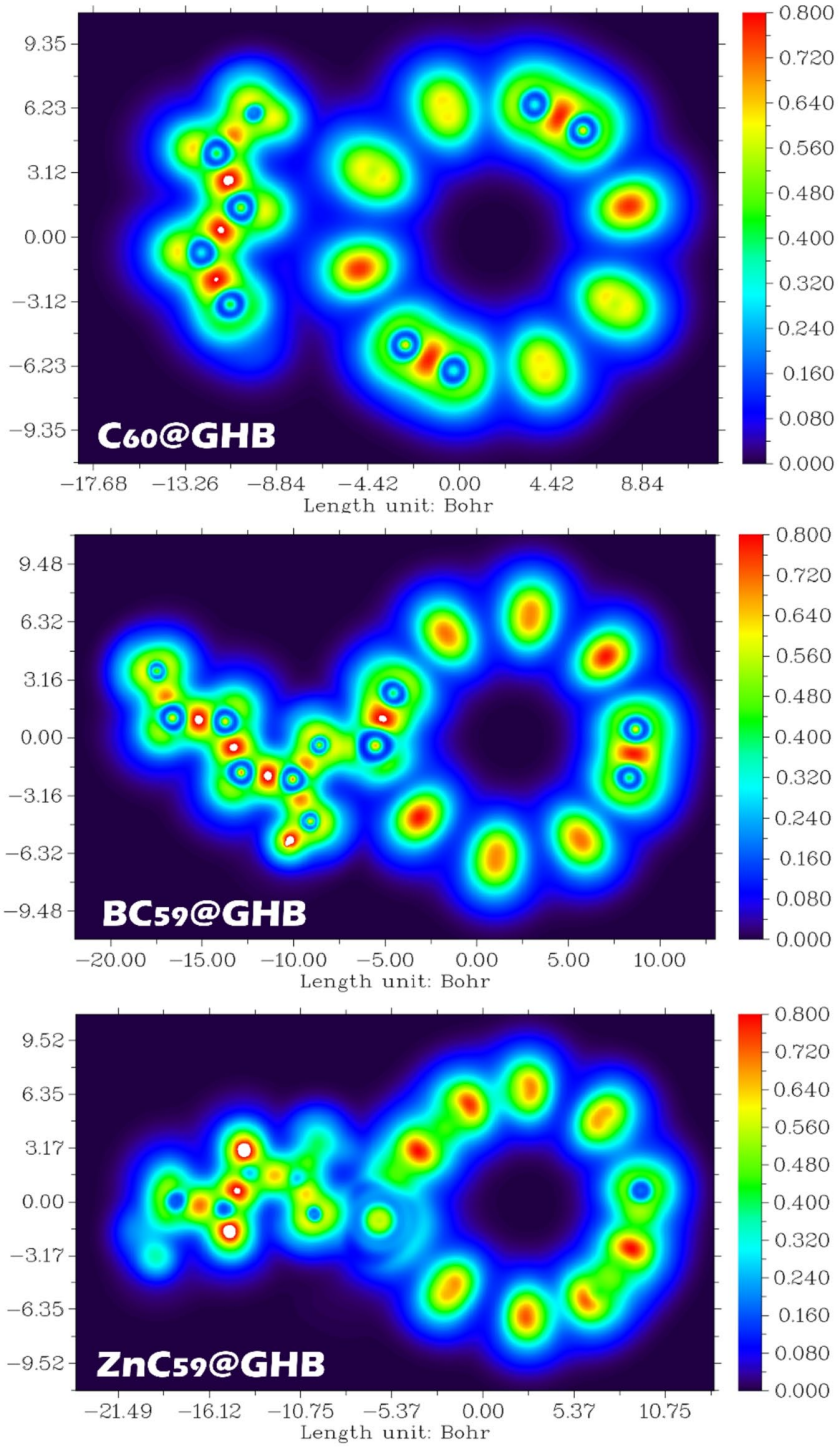
Local orbital locator (LOL) contour to investigate the gradual increase in electronic localization and charge accumulation at the host-guest interface for the C_60_@GHB, BC_59_@GHB, and ZnC_59_@GHB complexes.

In the LOL map (Fig. 16), electron localization is primarily confined to the intrinsic π-electron network of the C_60_ cage and the internal bonding regions of the GHB molecule. Only weak localization appears in the interfacial region, indicating minimal orbital overlap between the adsorbate and the pristine fullerene surface. This weak localization suggests that adsorption is dominated by dispersion forces rather than significant electronic interaction. For BC_59_@GHB, the LOL map exhibits enhanced electron localization in the vicinity of the adsorption site compared to pristine C_60_. Boron substitution perturbs the uniform π-electron distribution of the fullerene cage, creating localized regions of increased orbital activity near the interacting GHB molecule. This increased localization reflects stronger electronic coupling and polarization effects induced by doping. The ZnC_59_@GHB complex shows the most pronounced features in the LOL analysis. Strong localization is observed around the Zn center and extends toward the coordinating atoms of GHB, indicating significant orbital confinement and directional interaction. This localization is characteristic of metal-ligand coordination and suggests strong electronic coupling between ZnC_59_ and GHB. Such enhanced localization supports the strong adsorption behavior and identifies ZnC_59_ as a highly efficient system for GHB capture and removal.

**Fig. 17:**
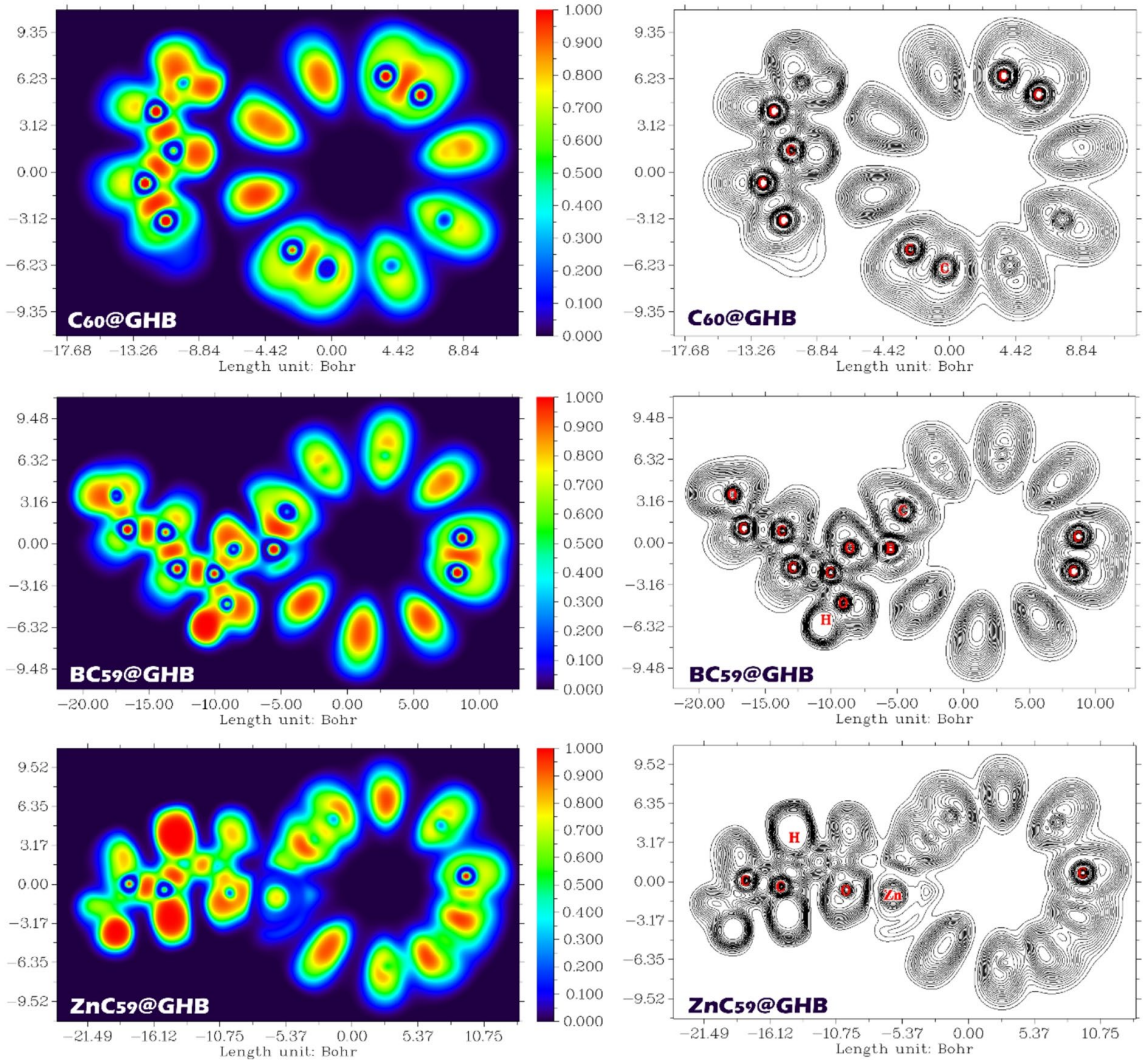
Electron Localization Function (ELF) contour maps (Color map on the left and black/white map on the right) of the optimized C_60_@GHB, BC_59_@GHB, and ZnC_59_@GHB complexes, illustrating the distribution of localized electron density and the evolution of host-guest interactions from weak physisorption in C_60_ to enhanced polarization in BC_59_ and strong metal-ligand coordination in ZnC_59_.

The Electron Localization Function (ELF) contours provide direct real-space insight into electron pairing, charge localization, and the nature of host-guest interactions in the C_60_@GHB, BC_59_@GHB, and ZnC_59_@GHB complexes. ELF values close to 1 (red regions) indicate highly localized electron pairs, whereas lower values (blue regions) correspond to delocalized or depleted electron density. Examination of these contours allows clear differentiation between weak physisorption and stronger chemically driven interactions (Fig. 17)^65^.

For the C_60_@GHB complex, ELF localization is largely confined to the intrinsic bonding regions of the C_60_ cage and the internal covalent bonds of the GHB molecule. The interface between GHB and C_60_ is characterized by low to moderate ELF values, with no continuous high-ELF basin bridging the two species. This absence of significant interfacial localization indicates minimal electron sharing and weak polarization effects, confirming that GHB adsorption on pristine C_60_ is governed primarily by van der Waals interactions. These features are consistent with weak adsorption strength and rapid desorption behavior.

In the BC_59_@GHB system, ELF contours reveal a clear enhancement of electron localization in the vicinity of the adsorption site. Boron substitution perturbs the uniform π-electron distribution of the fullerene cage, generating electron-deficient regions that promote stronger interaction with GHB. The presence of localized ELF basins at the interface indicates increased charge polarization and partial charge transfer between the adsorbate and the doped fullerene. Although no fully covalent bond is formed, the interaction is significantly stronger than in pristine C_60_, supporting the observed increase in adsorption energy and the pronounced sensitivity of BC_59_ to GHB adsorption.

Compared with every other system, ZnC_59_@GHB provided the most localized electrons based on the ELF contour map. The ELF contour map shows well-defined basins in the vicinity of zinc extending towards the oxygen atom in GHB, indicating that localized electrons are produced in greater quantities at the sites where zinc interacts with oxygen (in GHB) due to a strong charge pairing/stacking effect that demonstrates coordination between the metal and ligands. The evidence that supports the formation of the most stable chemisorption complex formed from the interactions of Zinc and Oxygen (in GHB) comes from the continuous ELF contour map between Zinc and GHB. Therefore, the evidence provided for localization of electrons between Zinc and GHB explains the incredibly high energy associated with the adsorption of ZnC_59_ from the GHB interface after the completion of adsorption. This finding, along with LOL and ELF analysis, is confirmed using NCI/RDG data and supports the conclusion that ZnC_59_ has better sensory efficiency than BC59 when used to detect or sense GHB.

### QTAIM

**Fig. 18:**
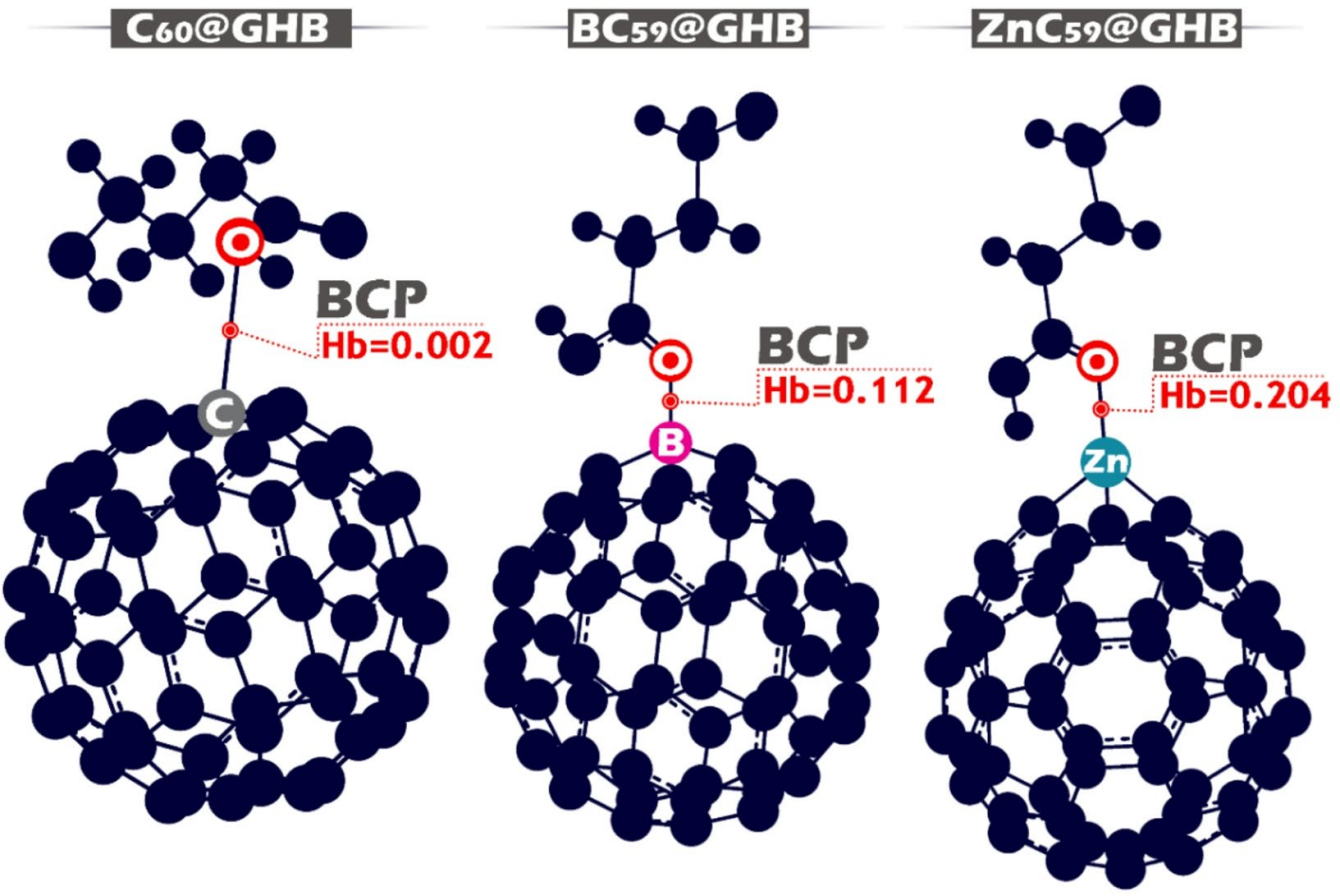
Comparative molecular models of GHB interacting with C_60_, BC_59_, and ZnC_59_ fullerenes, highlighting the bond critical points (BCP) and corresponding electron densities (Hb).

QTAIM analysis is a powerful framework for examining the nature of intermolecular interactions through the topology of the electron density at bond critical points (BCPs) (Fig. 18). Several topological parameters are commonly used within QTAIM to classify the strength and character of these interactions, and the classification of links follows the standard criteria of Bader. The electron density (ρ) at the BCP is a key indicator of interaction strength; higher values generally correspond to stronger interactions, whereas very low ρ values are characteristic of weak, noncovalent contacts. In accordance with Bader’s classification, interactions with low electron density at the BCP are typically associated with closed-shell interactions such as van der Waals forces, electrostatic attraction, or coordination effects rather than classical covalent bonding.

The Laplacian of the electron density (∇^2^ρ) provides information on charge concentration or depletion at the BCP. A negative Laplacian indicates local electron density accumulation, which may be observed in shared-shell interactions, whereas a positive Laplacian reflects electron density depletion and is characteristic of closed-shell interactions dominated by electrostatic or polarization effects^66,67^.

Additional insight is obtained from the analysis of energy density components at the BCP (Fig. 18). The kinetic energy density G(r) reflects electron localization effects, while the potential energy density V(r) represents stabilizing contributions to the interaction. Their sum defines the total energy density (Hb = V(r) + G(r)), which is widely used to distinguish interaction types. Negative Hb values indicate shared-shell or partially covalent interactions, whereas positive Hb values are indicative of closed-shell interactions governed by electrostatic, polarization, or dispersion forces^68^. Accordingly, in the present study, positive Hb values are interpreted as evidence of noncovalent or coordination-assisted interactions rather than strong covalent bond formation.

**Table 7:** Topological parameters at the bond critical point (BCP) for GHB interacting with C_60_, BC_59_, and ZnC_59_ fullerenes, including electron density ρ(r) (e.bohr^− 3^), Laplacian ∇^2^ρ(r) (e.bohr^− 5^), kinetic energy density G(r) (a.u.), potential energy density V(r) (a.u.), and the total interaction energy indicator VIR (a.u.).

Structure	ρ(*r*)	∇^2^ρ(*r*)	G(*r*)	V(*r*)	VIR
C_60_@GHB	0.0045	−0.0039	0.0032	−0.0007	0.00_24_
BC_59_@GHB	0.0705	−0.0706	0.0914	0.0208	0.1122
ZnC_59_@GHB	0.0952	−0.1022	0.1535	0.0512	0.2047

For C_60_@GHB, the small amount of electrons present at the BCP (ρ(r) = 0.0045 a.u.) indicates that there is a very weak bond between the two molecules. The slight negative Laplacian (∇^2^ρ(r) = −0.0039 a.u.) indicates that there is virtually no overlap of the electron density between these two molecules in the region of interaction. The positive kinetic energy density (G(r) = 0.0032 a.u.) combined with the small negative potential energy density (V(r) = −0.0007 a.u.) produces positive total energy density (Hb ≈ 0.002 a.u.) and an extremely small VIR (0.0024). Based on standard QTAIM criteria, these values indicate that the main force between C_60_ and GHB is through dispersion forces and not traditional covalent bonding. Consequently, the C_60_ and GHB interactions are weak physisorption, which is also supported by the NCI/RDG analysis showing mostly diffuse dispersion forces (Table 7).

For BC_59_@GHB, the value at the BCP for the associated electron densities was significantly increased, ρ(r) = 0.0705 a.u. indicating a much stronger bond relative to pristine C_60_. A negative Laplacian for the bond critical point, ∇^2^ρ(r)=−0.0706 a.u., indicates that charge concentration is enhanced in the interaction area. Although the total energy densities of the interaction are positive, both the potential energy, V(r) = 0.0208 a.u., and the kinetic energy, G(r) = 0.0914 a.u., which together lead to a positive total energy density, Hb = 0.112 a.u. (a relatively large value of the virtual internal resonance, VIR, 0.1122), does not definitively indicate the nature of the interaction as being a covalent bond. Instead, under the QTAIM approach outlined above, the parameters listed above clearly indicate that the interaction is dominated by an attractive electrostatic force and charge distribution, rather than covalent bonding. In this manner it can be said that, boron doping of carbon, increases charge redistribution and stabilizes boron doped GHB adsorption (as evidenced by the pronounced attractive region seen at the interface of the NCI/RDG contours).

ZnC_59_@GHB has the highest density of electrons at the BCP (ρ(r) = 0.0952 a.u.). ZnC_59_@GHB exhibits the greatest increase of total electronic density in comparison with the other systems evaluated, with a _max_imum potential value of the Laplacian of electronic density being negative (∇^2^ρ(r)= −0.1022 a.u.), indicating that a significant accumulation of electrons has been created in the region of interaction. Although the kinetic (G(r) = 0.1535 a.u.) and potential (V(r) = 0.0512 a.u.) energy densities are relatively substantial, the total energy density remains positive (Hb = 0.204 a.u.) thus yielding a _max_imum value of VIR (0.2047). Therefore, according to the Bader criterion, these characteristics represent an extremely strong closure shell interaction with high degrees of polarization and coordination in excess of that of a covalent bond. Therefore, the interactions associated with ZnC_59_ and GHB can be best described as that of a metal to ligand coordination whereby the Lewis Acid typifies the role of Zn. The NCI/RDG analysis supports this interpretation with evidence that localized large attractive regions of interaction exist on the interface of the ZnC_59_ and GHB systems.

**Fig. 19:**
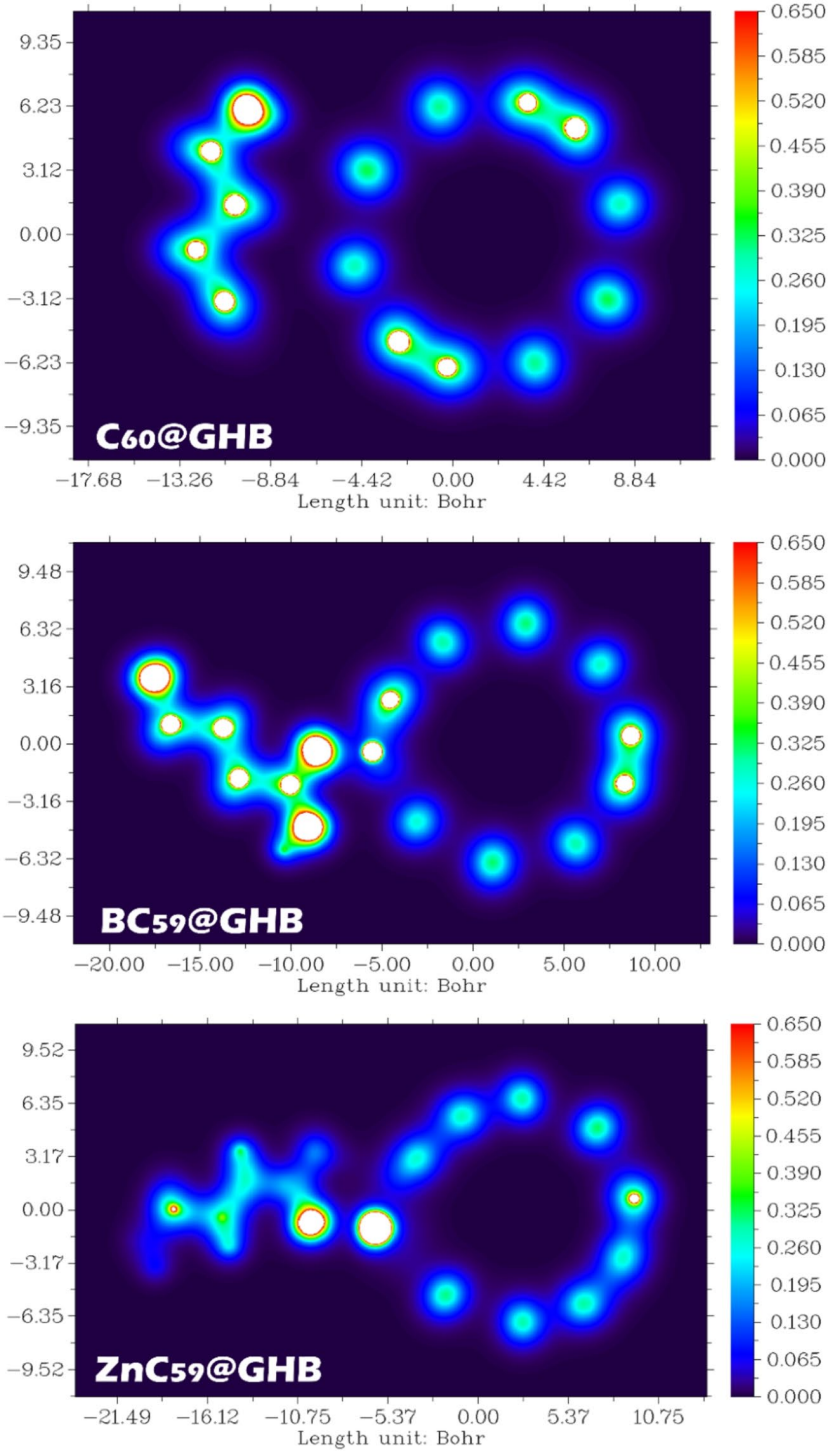
Two-dimensional electron density contour maps of the optimized C_60_@GHB, BC_59_@GHB, and ZnC_59_@GHB complexes.

It’s important to investigate electron density contours because they visually represent charge build-up and movement in the space where the sensor and sample are connected. For this reason, the type and strength of the interaction can be predicted (Fig. 19). By examining the continuity and electron density throughout the complex, it is possible to predict whether weak physical (indicated by discontinuous scattered density) or electrostatic (indicated by continuous dense electron density bridges) adsorption is present. Additionally, QTAIM parameters, like electron density at bond critical points, provide quantitative information about these types of interactions^69^.

According to the C_60_@GHB density distribution, electron density is primarily distributed over the delocalised π-rings in the fullerene (cage) and locations in GHB that have internal bonds. The region connecting C_60_ to GHB is weakly connected by a diffuse density that cannot be described as having a continuous connection (blue-green contours); thus, there has been very little electron density redistribution after GHB adsorbs onto C_60_. This is consistent with the QTAIM analysis, which shows low density at the bond-critical point (ρ ≈ 0.0045 a.u.) and a small negative Laplacian. These observations demonstrate that the interaction between GHB and pristine C_60_ is a result of weak van der Waals and dispersive interactions (as opposed to covalent bonds) with very little electron coupling.

As indicated by the electron density map for BC_59_@GHB, the charge accumulation at the point of adsorption has a significant increase. The regions near the point where GHB comes in contact with the BC_59_ exhibit an increase in both intensity and area. This indicates that the boron dopant is inducing a greater degree of polarization and partial charge transfer. The density remains localized at the BC_59_@GHB binding contact (BCP), but it is clearly much higher in magnitude than that of the C_60_@GHB (the associated negative Laplacian is also increased). This increased density and decreased negative Laplacian provides evidence of an increased degree of closed-shell electrostatic interaction between the GHB and the boron doped C_60_, along with a large increase in polarization driven interaction, both of which are responsible for the increase in adsorption energy, as well as an increased response for sensing capabilities exhibited by BC_59_.

The electron density map of ZnC_59_@GHB has the most distinct features compared to all other systems studied. There is a continuous and strong density bridge visible between the metal ion and the GHB molecule, and there is an abundance of bright yellow/red contours at the site of interaction where charge is concentrated, showing directional bond formation typical for metal-coordination complexes. This visual representation also correlates with the QTAIM results that present very high densities at the bond critical point (ρ = 0.0952 a.u.), and the lowest Laplacian value of all systems studied. The combination of these topologically derived parameters supports the observation that there is a very strong, stabilized interaction between ZnC_59_ and GHB characterized by partial covalency/coordination; thus, ZnC_59_ adsorbs GHB more effectively than other metal-organic frameworks investigated.

The QTAIM results confirm the previous results obtained through NCI/RDG, ELF and LOL analyses and verify that ZnC_59_ is the best adsorbent and BC_59_ is an excellent but comparatively weakly-interacting system. Conversely, C_60_@GHB has been found to exhibit only weak non-covalent interactions with the substrate.

## Conclusion

In this work, a systematic in silico investigation was performed to assess the suitability of pristine and doped fullerene-based nanostructures for the adsorption, sensing, and removal of gamma-hydroxybutyric acid (GHB). All calculations were carried out within the framework of Density Functional Theory (DFT) using the B97D functional with the 6-31G* basis set and the CPCM solvation model to represent aqueous conditions. The reliability of the chosen functional is supported by the strong overlap between the calculated electronic properties of pristine C_60_, particularly the HOMO-LUMO gap (1.67 eV), and experimentally reported values (~ 1.50–1.86 eV), thereby justifying its use for predicting the behavior of fullerene-based sensor systems. Qualitatively, pristine C_60_ exhibits weak interaction with GHB, as reflected by its relatively small adsorption strength (−14.03 kcal.mol^− 1^), negligible conductivity variation (≈ 2.20 × 10^9^ to 2.21 × 10^9^ S.m^− 1^), and very short recovery time (1.94 × 10^− 2^ s). These values collectively indicate physisorption governed mainly by dispersion forces, which limits the effectiveness of pristine C_60_ for reliable sensing or capture of GHB. Boron doping substantially improves sensor performance. The BC_59_@GHB system shows stronger adsorption (−23.11 kcal.mol^− 1^), a notable reduction in the HOMO-LUMO gap upon adsorption (from 1.35 to 0.98 eV), and a clear enhancement in electrical conductivity (2.35 × 10^9^ to 2.53 × 10^9^ S.m^− 1^), accompanied by a long recovery time (1.51 × 10^5^ s). Qualitatively, these trends indicate enhanced charge transfer and stronger electronic perturbation, making BC_59_ particularly favorable for electrochemical sensing applications where signal modulation is critical. Zn-doped fullerene exhibits the strongest interaction with GHB. ZnC_59_@GHB displays the most pronounced adsorption strength (−26.91 kcal.mol^− 1^), an intrinsically small energy gap (0.36 eV), and an extremely long recovery time (4.27 × 10^7^ s), indicating very stable adsorption. Although conductivity changes are modest (2.87 × 10^9^ to 2.85 × 10^9^ S.m^− 1^), the qualitative behavior clearly identifies ZnC_59_ as an efficient adsorbent rather than a reversible sensor.

The bonding nature and interaction strength were further elucidated through QTAIM, NCI/RDG, ELF, and LOL analyses. QTAIM results reveal a very low electron density at the bond critical point (ρ = 0.0045 a.u.) for C_60_@GHB, confirming weak van der Waals interactions. In contrast, BC_59_@GHB shows a significantly higher ρ value (0.0705 a.u.), while ZnC_59_@GHB exhibits the highest electron density (0.0952 a.u.) and the most negative Laplacian, indicating strong charge accumulation and partial covalent/coordination character. These findings are fully consistent with the NCI/RDG analysis, where C_60_@GHB is dominated by diffuse green isosurfaces (weak dispersion), BC_59_@GHB displays mixed green-blue regions (enhanced electrostatic attraction), and ZnC_59_@GHB shows intense blue regions corresponding to strong attractive interactions. ELF and LOL contour analyses further corroborate these conclusions by demonstrating progressively enhanced electron localization and interfacial charge redistribution in the order C_60_@GHB < BC_59_@GHB < ZnC_59_@GHB. Weak interfacial localization in pristine C_60_ confirms physisorption, whereas pronounced localization around boron and especially zinc centers highlight polarization effects and metal-ligand coordination as the dominant stabilizing mechanisms in the doped systems.

It is important to emphasize that the numerical values reported in this study are primarily used as indicators to predict relative sensor response rather than as absolute experimental quantities. In computational sensor design, qualitative trends (such as the systematic increase in adsorption strength, charge transfer, and electronic perturbation from C_60_ to BC_59_ to ZnC_59_) are more meaningful than the exact magnitudes of individual parameters. Therefore, evaluating sensor behavior based on the consistent trends observed across multiple descriptors provides a reliable framework for predicting practical performance. Based on these qualitative trends, BC_59_ emerges as the most promising candidate for GHB sensing applications, while ZnC_59_ is better suited for GHB adsorption and removal. The results of this computational study are thus recommended as a guiding platform for future experimental and laboratory investigations aimed at developing efficient, low-cost, and rapid fullerene-based nanosensors and adsorbents for GHB detection.

## Data Availability

All data generated or analyzed during this study are included in this published article.
